# Bonding Analysis of Compounds with Unusual Coordination of Carbon: Proposed Symmetric Systems with Six-Coordinate Carbon

**DOI:** 10.3390/molecules25173937

**Published:** 2020-08-28

**Authors:** Carl Trindle, Zikri Altun, Erdi Ata Bleda

**Affiliations:** 1 Chemistry Department, University of Virginia, Charlottesville, VA 22904, USA; 2 Physics Department, Marmara University, Istanbul 34724, Turkey; zikalt@marmara.edu.tr (Z.A.); ata.bleda@marmara.edu.tr (E.A.B.)

**Keywords:** AIM modeling, electron localization function, non-covalent interaction, carbon hypercoordination

## Abstract

The possibility of carbon tetravalence in geometries other than tetrahedral and of carbon hypervalence has been taken seriously since the 1970s. Computational modeling and subsequent experimental validation have established the existence of molecules with carbon atoms with planar tetravalence and as many as six objects in carbon’s coordination sphere. In this work, we develop insight into the nature of bonding to carbon in these unusual environs as provided by Bader’s Atoms in Molecules (AIM) analysis of the electron density, along with the electron localization function (ELF) and the non-covalent index (NCI). We review several well-established systems (spiropentadiene dication, hexamethyl benzene dication, dimethanospiro[2.2]octaplane dication, and 1,8-dimethoxy-9-dimethoxyanthracene cation) and propose new D_2d_–symmetric variants of a hexacoordinated species.

## 1. Introduction

One of the basic insights of organic chemistry is that saturated carbon atoms arrange four partners in tetrahedral coordination. [1,2,3,4] Lewis electron pairing and the Valence Shell Electron Repulsion model [5,6] reinforce the insight, allow easy applications, and shape our intuition. Minor departures from the ideal form can be interpreted as a consequence of the lower symmetry of the arrangement of the four objects in the coordination sphere. But major departures from tetrahedral tetracoordination—that is to say, either serious change as from (a) tetrahedral to planar four-coordination or (b) hyper-coordination with five or more neighbors—are rare, and require special explanation.

**Figure 1:**
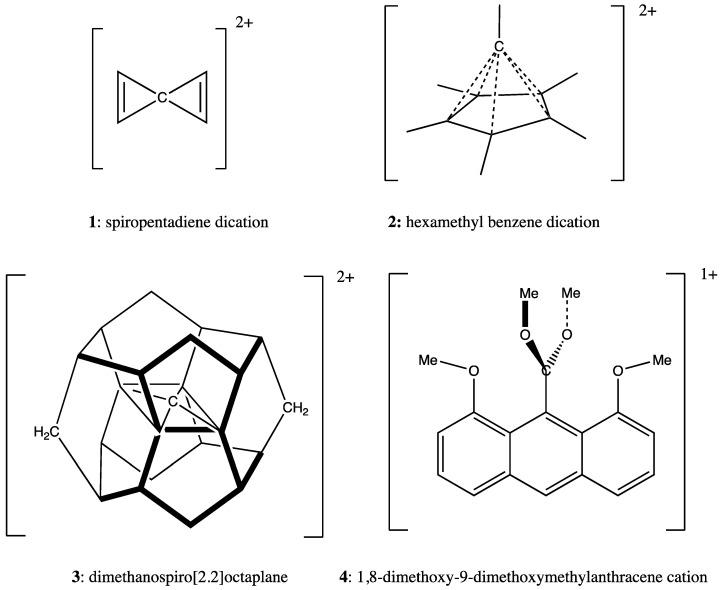
Notable structures with unusual coordination of carbon.

The instability of planarized tetracoordinate carbon as in planar methane (called tpC,) is attributed to its nonbonding pi lone pair [7,8]; in the tetrahedral form all four valence pairs participate in bonds. Planarity can be enforced by two kinds of influence: (1) Removal of the pair of electrons that destabilize the planar form, first exemplified in the discussion of planar methane [7]; (2) ring strain; and/or (3) suitable substitution and associated electronic effects. For example, the closed-shell ground state dication of CH_4_ is planar (qualitatively, a C_2v_ complex of H_2_ with CH_2_ dication) [9]; the double ionization removes charge from the p-pi AO of the Carbon. We discuss structures for other dications with unusual coordination of a central carbon atom, notably **1**–**4** (Figure 1). The dication of spiropentadiene (**1**) is D_2h_ in symmetry, as modeled computationally by Lammertsma and Schleyer [10]. The pentagonal-pyramidal structure of hexamethylbenzene **2** is achieved only in the dication, established experimentally by Hogeveen et al. [11,12,13].

**Figure 2:**
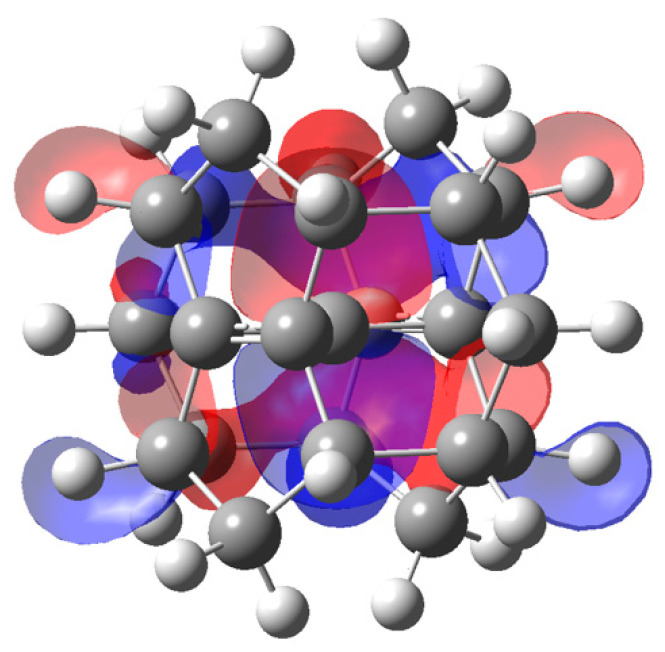
HOMO for neutral dimethanospiro[2.2]octaplane.

Radom’s alkaplane systems [14] rely entirely on strain to force the central CC_4_ fragment toward planar tetracoordination of Carbon. The tpC with its immediate C neighbors in D_2h_ dimethanospiro[2.2] octaplane **3** is shown in Figure 1. However, the stability of the neutral D_2h_-symmetric form is questionable. Radom and Rasmussen [14] report that a MP2/6-31G(d) calculation (by Mark S. Gordon, unpublished) produces all-real vibrational frequencies, suggesting the D_2h_ structure occupies a relative minimum on the potential surface. B3LYP/6-31G(d) calculation and our ωB97XD/cc-pVTZ calculation both produce a substantial imaginary frequency (ca. 300i) suggesting the symmetric form is a transition state between two twisted forms of the CC_4_ fragment in the dimethanospiro[2.2]octaplane. Figure 2 displays the isosurface of the HOMO for neutral **3**, which is dominated by the troublesome C lone pair. This form of the HOMO is in general agreement with results of calculations at lower levels of theory [14,15].

The planarity can be established by ionization; the monocation radical has a planar tpC [15]. We have confirmed that complete removal of two electrons from the HOMO more firmly establishes stability of the D_2h_ symmetry of Radom’s dimethanospiro[2.2]octaplane dication.

Substitution of two Carbon atoms by Boron atoms, forming systems isoelectronic with the hydrocarbon dications, accomplishes planarization. This formation of neutral analogs to hydrocarbon cations is called the charge compensation principle by its inventors [13] and is applied to this issue by Wang and Schleyer [16]. A tactic recommended by Hoffmann [7,8] is to surround the central carbon by species which are strong sigma donors and pi acceptors, which then may effect redistribute the troublesome planar-Carbon lone pair toward its surroundings. A series of molecules incorporating Copper and Nickel substituents on the tpC investigated by Schleyer and Boldyrev [17] reverse this motif, calling for strong pi-donor and sigma-acceptors on a periphery. A central example is CAl_4_ anion [16]. Structures with many other metals have been evaluated computationally [18,19,20,21].

The same stratagem has prompted computational studies of planar penta- and hexa-coordinated Carbon. For example, Exner and Schleyer [22] used B3LYP and CCSD(T)/6-311+G(d,p) to describe a planar CB_6_^2−^ system as well as isoelectronic and neutral C_3_B_4_ and other variants.

In this report we confine our attention to metal-free systems and examine hypercoordinated Carbon atoms with O and S members in the coordination sphere. The coordination sets in our systems are often nonplanar. Our purpose is to characterize the interactions of the hypercoordinated carbon with its neighbors. To assist intuition we employ diagnostics and visualization aids from Bader’s Atoms-in-Molecules theory [23], and related quantities such as the electron localization function [24] and the non-covalent index [25].

## 2. Computational Methods and Software

### 2.1. Software

We use Gaussian 09 [26] and 16 [27] for electronic structure calculations including geometry optimization and vibrational analysis. The density functional model is ωB97XD [28] and the basis is cc-pVTZ [29] except as otherwise noted. With one exception (**5**) in which the experimental (X-ray) structure is used, all reported structures are local minima according to ωB97XD/cc-pVTZ, with no imaginary vibrational frequencies. Bader AIM analysis is conducted by AIMALL [30] and Multiwfn software [31]. Construction of the electron localization function (ELF) and the non-covalent index (NCI) is accomplished with Multiwfn and NCIplot [32]. In the following sections we describe AIM, ELF, and NCI methods of characterizing the electron density. Those familiar with these analyses may wish to pass directly to Section 3, in which we describe applications.

### 2.2. AIM and Bader Terminology

The Atoms-in-Molecules (AIM) analysis characterizes bonding with reference to the electron density, an observable of a molecular system. The review by Kumar et al. is a useful introduction, with many examples [33]. At critical points where ∇*ρ* = 0 we can evaluate the charge density *ρ*, its Laplacian *L* = ∇^2^*ρ*, and the eigenvalues {λ_k_} of the matrix {H_ij_} = {∂^2^*ρ*/∂x_i_∂x_j_} along with various energy densities. Diagonalization of **H** yields three eigenvalues. There can be three negative eigenvalues, defining NCP nuclear critical points (attractors) coded (3, −3); two negative and one positive value defining BCP bond critical points, coded (3, −1), one negative and two positive values defining RCP ring critical points coded (3, +1), or three positive values defining CCP cage critical points coded (3, +3). The number of critical points of all kinds should obey the Poincare-Hopf rule, which in this case requires that NCP − BCP + RCP − CCP = 1. This is the case for all systems described here.

AIM divides Cartesian space into basins, each containing an attractor—most often a nucleus. The boundaries of the basins are defined by a zero-flux (∇*ρ* = 0) surface. Integration of electronic charge within a basin including a nucleus produces an AIM atomic charge.

For familiar covalent chemical bonds, the electron density *ρ* at a BCP would be on the order of tenths of density units, electrons per cubic bohr. For weak mainly electrostatic interactions such as H bonds, the density at the BCP would be much smaller (at best, hundredths of atomic units) and the Laplacian would be positive. In all the examples to follow, we find the usual array of nuclear attractors with very large densities and large negative Laplacian values; BCPs for covalent sigma bonds; nonpolar aromatic CC bonds in the anthryl rings; and polar bonds with smaller densities. In AIM analysis the kinetic and potential energy densities G and V at the bond critical points offer insight into the nature of bonding. A useful diagnostic of covalent bonding is the ratio |2G(**r**_bcp_)/V(**r**_bcp_)|; if this ratio is less than unity then the interaction is covalent [34].

While AIM provides a well-defined methodology for processing molecular electron density (obtained either by theoretical methods or by processing experimental data) its link to intuitive chemical constructs—especially bonds and atomic charges—has been the subject of considerable debate. We refer to only a very small sample, which will convey the flavor [35,36,37,38]. We judge that *reductio ad absurdum* does not undermine the practical value of QTAIM.

### 2.3. ELF

The electronic localization function (ELF) was defined by Becke and Edgecombe [24]. The probability density that a second like-spin electron is found near a “localized” electron is presumably small. This density arising from Fermions in the molecular system is written as:

(1) $$D=\Sigma_{i}^{\sigma }{\left|\nabla \psi_{i}\right|}^{2}-{\left|\nabla \rho \left(r\right)\right|}^{2}/4\rho \left(r\right)$$

The sum is over all the occupied molecular spinorbitals; $\rho_{\sigma }\left(r\right)$ is the electron density of electrons with σ spin which in the Kohn–Sham approximation is given by $\rho_{\sigma }\left(r\right)=\Sigma_{i}^{\sigma }{\left|\psi_{i}\right|}^{2}$. The smaller the quantity *D*, the more highly localized is the reference electron. A dimensionless index χ is defined as the ratio of the density *D* to the corresponding value for the uniform electron gas:

(2) $$D_{0}=\frac{3}{5}{\left(6\pi^{2}\right)}^{2/3}\rho^{5/3}.$$

ELF is confined to the range [0, 1] by the transform ELF = 1/(1 + χ^2^). The limit of maximum localization is χ = 1.

### 2.4. NCI

A useful description of the non-covalent index, its graphical characterization, and its interpretation is given by Contreras-Garcia, et al. [39]. The reduced (dimensionless) density gradient *s* (also called RDG) is defined as

(3) $$s\left(r\right)=\frac{\left|\nabla \rho \left(r\right)\right|}{2{\left(3\pi^{2}\right)}^{1/3}\rho \left(r\right)r^{4/3}}$$

**Figure 3:**
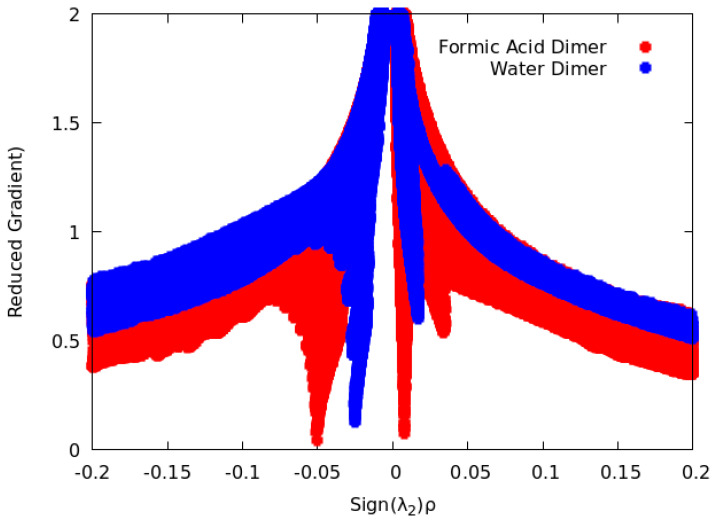
Two-dimensional representation of non-covalent interactions for water dimer and formic acid dimer.

Two NCI graphic realizations are useful. A two-dimensional plot of *s* vs. the density *ρ* (given the sign of the second eigenvalue of **H**) will produce a broad sweep from one extreme at small density but large *s* to large density and small *s*. This is characteristic of an exponentially-decaying density. Superimposed on this sweep may be spikes in *s* extending to values near zero at modest values of density. In Figure 3, plots derived from ωB97XD/cc-pVTZ computations for water dimer and formic acid dimer are shown. The high-density (but low *s*) regions near nuclei are far to the right or left of the range of densities shown. The low-density region is located primarily in Cartesian space close to the density tails for which *s* is large. However, *s* can be small precisely where the density is disturbed by non-covalent interactions, such as van der Waals/dispersion and electrostatic forces. Figure 3 shows the plot for water dimer and formic acid dimer. For water dimer, we find one such case at signed density about −0.025, and for formic acid dimer we find two, at +0.01 and −0.05.

**Figure 4:**
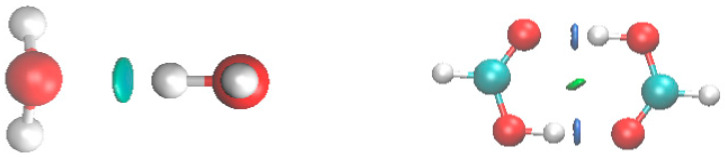
Zones for which both reduced density gradient (RDG) and density are small for water dimer (L) and formic acid dimer (R).

Mapping those zones of small density and small *s* (RDG) into three-dimensional space (Figure 4), we isolate regions of significant NCI in isosurfaces of value 0.05. For water dimer there is a single zone of non-covalent interaction, which we must associate with H-bonding. One spike from Figure 3 can refer to more than one zone. In the formic acid dimer (Figure 4), we find two attractive H-bonding NCI regions and also a destabilizing zone corresponding to a ring critical point.

## 3. Density Analysis for Known Examples of Unusual Coordination of Carbon

### 3.1. Example 1: Spiropentadiene Dication

Our ωB97XD/cc-pVTZ calculations and earlier reports [10] verify that D_2h_-symmetric spiropentadiene dication **1** occupies a relative minimum energy point. (The unsubstituted monocation and neutral counterparts however are nonplanar.) The neutral system can be constrained to coplanarity of the three-membered rings by enclosing the spiropentadiene core within aromatic seven-membered rings [40]. In this case the troublesome electron pair is exported from the p-pi AO of tpC into the aromatic region.

#### 3.1.1. AIM Analysis

**Figure 5:**
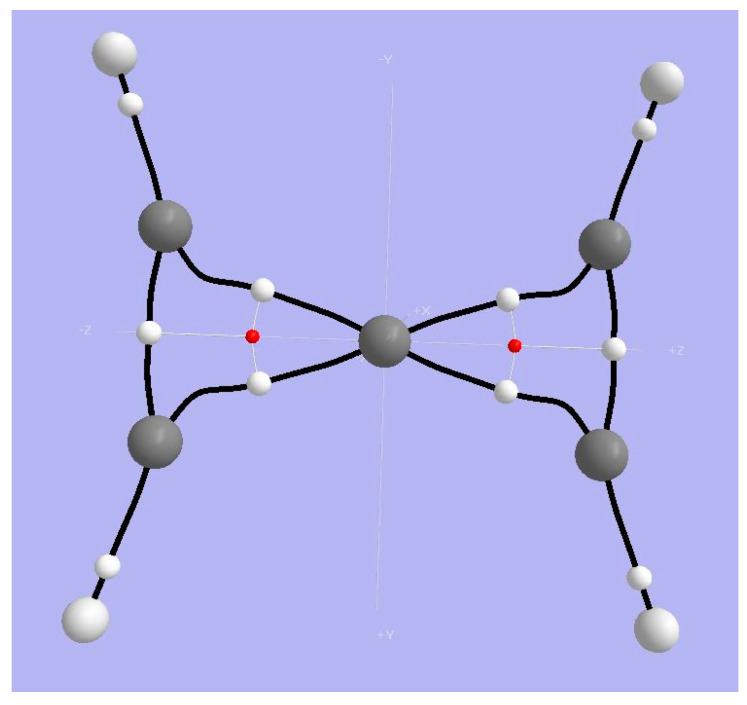
Topological (Bader) analysis of spiropentadiene dication **1**. Large light-gray spheres = H atoms; large dark gray spheres = C atoms; small white spheres = BCPs; small red spheres = RCPs; black lines = bond paths.

Firme et al. [41,42] presented an AIM analysis of the planar dication of spiropentadiene. Critical points and bond paths from our AIM2000 analysis of the density obtained with ωB97XD/cc-pVTZ are shown in Figure 5. Values of density, the Laplacian, G and V at selected BCPs are shown in Table 1. Entries in red are from Firme, et al. [41] and agree pretty nearly with our ωB97XD/cc-pVTZ values. We see that the BCPs along the paths from a peripheral Carbon to the tetracoordinate planar Carbon have much smaller values of the Laplacian of the density compared with the values at BCPs along the two bond paths connecting peripheral C-Cs. We infer that the tpC-Cx bond is weaker than the Cx-Cx bond. It is remarkable that the tpC is has a net negative AIM basin charge (−0.193 absolute electrons or |e|); the positive charge in this dication is dispersed to the surrounding C and H atoms (+0.169 and +0.379 |e| respectively.)

#### 3.1.2. ELF Analysis

**Figure 6:**
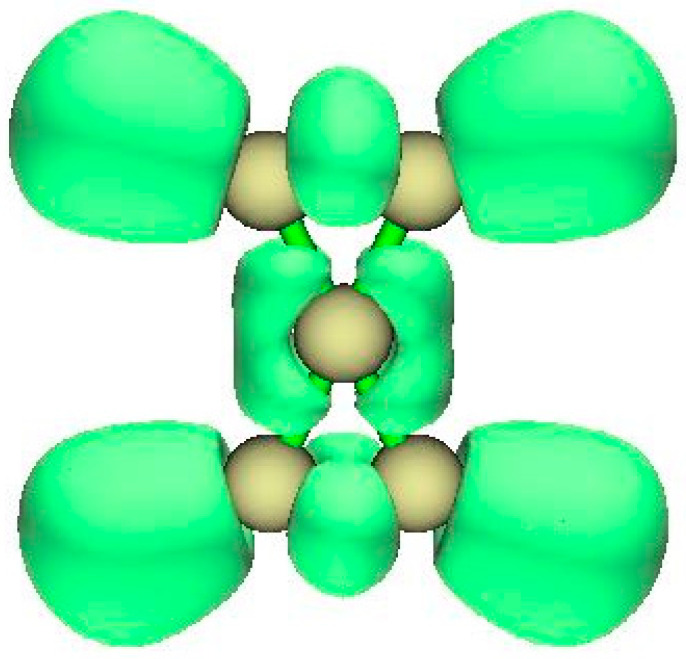
Electron localization function (ELF) for spiropentadiene (view perpendicular to D_2h_ plane).

The electron localization function shown in Figure 6 displays the four bonding regions C to H and the two single CC bonds, top and bottom. The pi character of the C=C bonds is visible at the bottom. The ELF zones for the CH bonds are extensive, compared with CC bonds. From the AIM basin charges, (Table 1) it appears that upon double ionization electron density from the HC=CH fragments has migrated so that tcC obtains a net negative charge.

#### 3.1.3. NCI Analysis

The plot of the reduced density gradient *s*(*ρ*) against the density as signed by the second eigenvalue of the Laplacian of the density shows no departures from monotone decline in *s*(*ρ*) at left and right (See Figure S1, Supplementary Materials). That is, there are no zones of non-covalent interaction in this dication.

### 3.2. Example 2: Hexamethylbenzene Dication

The structure of hexamethylbenzene dication **2** found by ωB97XD/cc-pVTZ is a pentagonal pyramid, in which a -CMe cap subtends a five-membered ring. The carbon of the cap could be considered hexacoordinate, and termed hcC. The crystal structure has been reported [43] along with proton and C-13 NMR. Bonding is characterized by NICS [44] the system displays three-dimensional aromaticity. Apical to basal CC bond orders are small (ca. 0.5), and the apical (hexa-coordinate) Carbon employs only four electrons in bonding. That is, the octet rule is not violated.

#### 3.2.1. AIM Analysis

**Figure 7:**
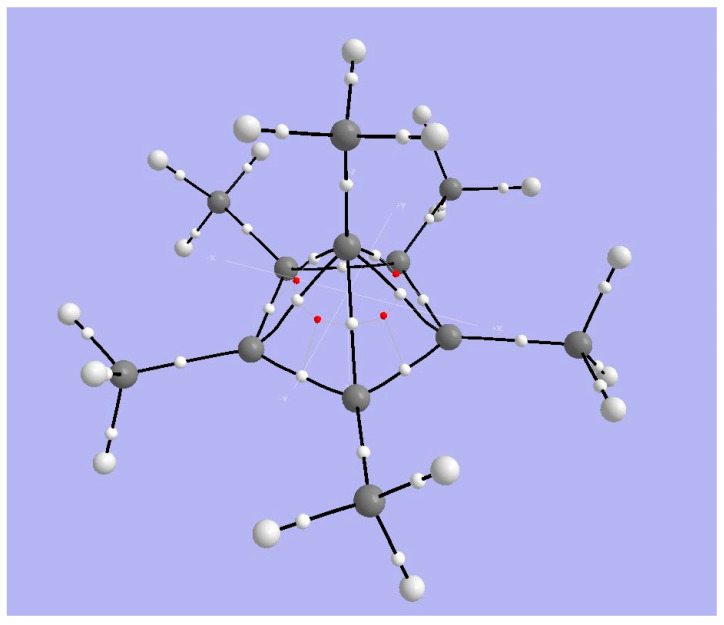
Topological (Bader) analysis of hexamethylbenzene **2**. Large light-gray spheres = H atoms; large dark gray spheres = C atoms; small white spheres = BCPs; small red spheres = RCPs; black lines = bond paths.

Figure 7 shows bond critical points and bond paths reinforcing the concept that the cap -CCH_3_ interacts with all five of the carbons in the pentagonal base. Similarly, CC bond paths define bonding between neighboring C atoms in the pentagonal base and their attachment to methyl groups. Each face of the pentagonal pyramid contains a ring critical point (RCP).

The values of the Laplacian at AIM bond critical points (shown in Table 2) suggest that the C (ring or apex) to C (methyl) bonds are conventional single bonds (the Laplacian is about −0.5, density about 0.27) while the CC bonds within the ring are appreciably stronger, (the Laplacian is about −0.8, density about 0.28). The BCPs for the bonds from apical C to ring Cs are distinct; density is about half that of the single covalent bonds just mentioned and the Laplacian >0, suggesting that charge is expelled from that region, covalency is weak, and there is some electrostatic component to the interaction. Klein, Havenith, and Knizia [45] consider the compound to be best characterized as a donor-acceptor complex of anionic cyclopentadiene and a “highly Lewis-acidic Carbon atom… capable of acting as an electron-pair donor to a formal CH_3_^+^ group.” This is an appealing way to describe the bonding partly because it lends itself so well to interpretation of bonding by means of an interaction diagram. The result of charge migration, as expressed in the basin charges of AIM, is to produce the base ring of five C atoms with associated basin charges near 6.02 electrons (|e|) on average, with an outer ring of five methyl C atoms with basin charges near 6.037 on average. The basin charge corresponding to the apical six-coordinate C atom is 6.088, and its methyl C basin charge is 6.016 (|e|). That is, even in this dication the C frame is net negative in charge. The positive charge making up the net +2 is borne by H atoms.

#### 3.2.2. ELF Analysis

**Figure 8:**
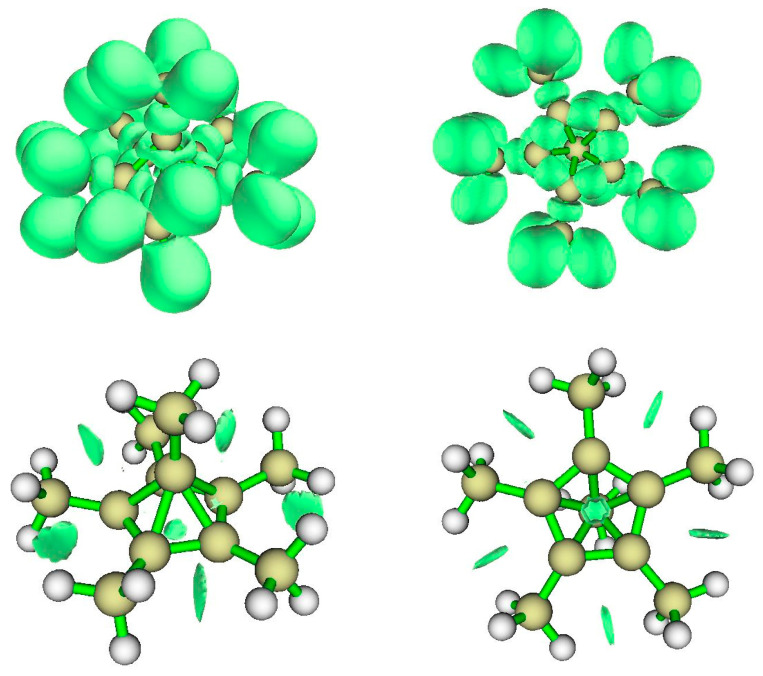
Views of the electron localization function isosurfaces (**top**) and the non-covalent interaction zones (**bottom**); views from an elevation toward a face of the pentagonal pyramid (**left**) and from below the base (**right**).

Figure 8 displays the electron localization function (ELF) surface from an elevation toward a face of the pentagonal pyramid (top left) and a view from the pentagonal base (top right). The former view reveals a bond connecting the apical carbon to the carbon of its methyl, and the region of the bonding around the carbon five membered ring. The latter view shows base CC bonds, bonds from those base-plane Carbons to their methyl carbons, and the visible portions of the associated CH bonds. The apical C atom is visible in the center.

#### 3.2.3. NCI Analysis

Figure 8 (bottom) displays non-covalent interaction zones for hexamethyl benzene dication. These include repulsions between adjacent methyl groups in the (CH_3_C)_5_ base ring, and a feature at the center of that ring. The plot of the reduced density gradient (RDG) *s*(*ρ*) vs. the signed density (Supplementary Materials) suggests that the NCI zones identify two kinds of weak van der Waals interactions, which we can see are the ring methyl-methyl interactions and the NCI zone at the center of the five-carbon ring.

### 3.3. Example 3: Dimethanospiro[2.2]octaplane

To our knowledge there is no experimental realization of the C_5_H_4_ dication **1** itself, but Radom’s ways of stabilizing this structure by steric constraint and charge delocalization might point the way toward a synthesis. The dimethanospiro[2.2]octaplane dication **3** discussed above contains a tetracoordinate planar Carbon (tpC) similar in structure to the spiropentadiene dication. The central CC_4_ fragment is forced toward planarity primarily by the surrounding rigid cage structure. The constraint is most effective for the dication, and the stable minimum has D_2h_ symmetry

#### 3.3.1. AIM Analysis

**Figure 9:**
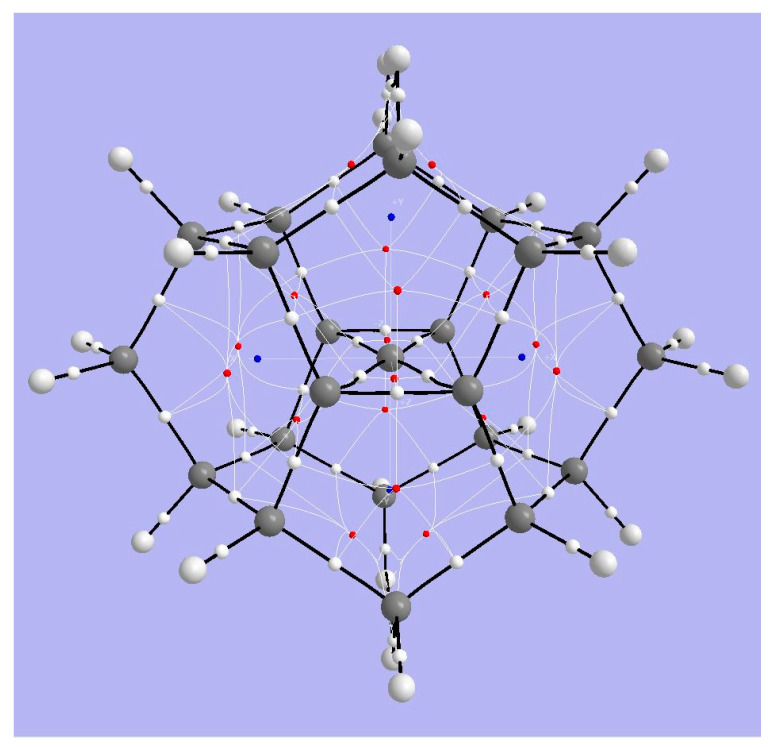
Topological analysis of Radom’s dimethanospiro[2.2]octaplane **3**. Large light-gray spheres = H atoms; large dark gray spheres = C atoms; small white spheres = Bond CPs; small red spheres = Ring CPs; small blue spheres = Cage CPs; black lines = bond paths.

Figure 9 displays bond critical points, (white), ring critical points (red) and cage critical points (blue). The Bader parameters for cage CC and CH bond critical points (Table 3) are characteristic of saturated hydrocarbons. Note that there are bond critical points and bond paths between two pairs of C atoms coordinated to the tpC, consistent with the rectangular arrangement and the overall D_2h_ symmetry, as in spiropentadiene dication **1**. As in the case for **1**, the tpC has a negative AIM basin charge (−0.165 |e|); the positive charge in this dication is dispersed not substantially to the surrounding C atoms in the C_5_ plane (+0.028 |e| each) but rather mainly to the constraining cage atoms.

#### 3.3.2. ELF Analysis

**Figure 10:**
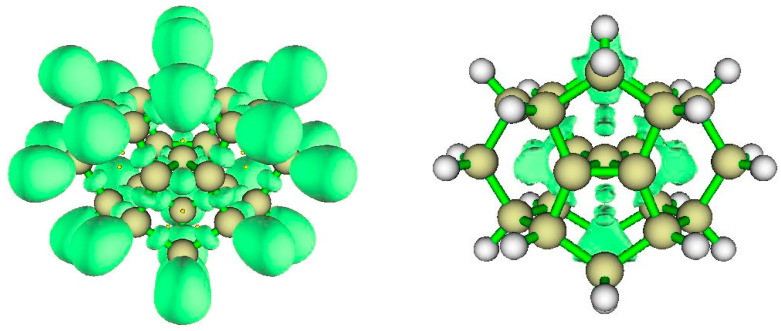
ELF for Radom’s D_2h_ dimethanospiro[2.2]octaplane **3**, (**left**) showing central tpC and its bonds to its neighbors. The NCI zones (**right**) reveal the non-covalent interactions within the rings.

The ELF isosurface diagram shown in Figure 10 (left) reveals the essentially covalent character of the bonds of tpC to its neighbors and the absence of localized charge perpendicular to the plane containing the tpC and its four bonded C atoms. The lack of localized pi charge at tpC is the consequence of double ionization to the dication.

#### 3.3.3. NCI Analysis

The plot of the reduced density gradient (RDG) *s*(*ρ*) vs. the signed density (Supplementary Materials) shows van der Waals features and also strongly repulsive interaction. The NCI zones shown in Figure 10 (right) lie at cage critical points and between the >CH_2_ bridges of the cyclooctane fragments at top and bottom of the structure. Curious NCI zones appear above and below the CC4 central plane.

### 3.4. Example 4: 1,8-Dimethoxy-9-Dimethoxymethylanthracene Cation

Akiba and co-workers [46,47,48] have reported synthesis and isolation of a compound with roughly trigonal bipyramidal five-coordinated carbon (tbpC). The C_2v_-symmetric structure from ωB97XD/cc-pVTZ is shown in Figure 1 as species **4**. The X-ray analysis shows that the distance between the Anthryl methoxy Oxygen and the cationic 5-coordinated tbpC (2.44 ± 0.01 A) is smaller than the distance between the ipso carbons of anthracene to which they are attached (2.50 ± 0.02 A). Computational characterization [40] of the system also reflects a shortening, which suggests an attractive MeO…tbpC electrostatic interaction.

#### 3.4.1. AIM Analysis

**Figure 11:**
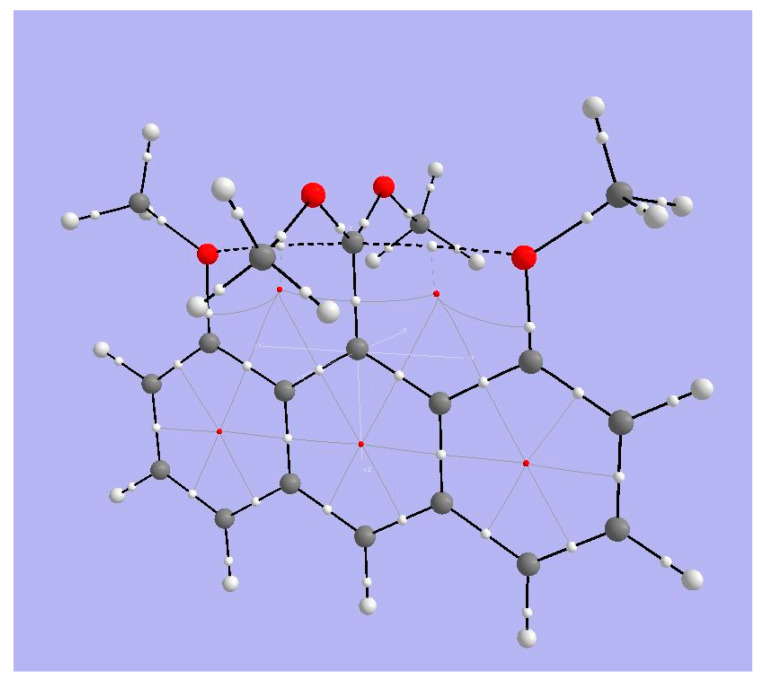
Topological analysis of Akiba’s five-coordinate Carbon compound **4**. Large light-gray spheres = H atoms; large dark gray spheres = C atoms; small white spheres = Bond CPs; small red spheres = Ring CPs; solid black lines = bond paths; broken black lines = bond paths for weak interactions.

Figure 11 shows BCPs and RCPs for 1,8-dimethoxy-9-dimethoxymethylanthracene cation (species **4** in Figure 1). The broken lines representing bond paths connecting anthryl-methoxy oxygens and the tbpC indicate a weak interaction. The Bader parameters for BCPs (Table 4) bear out the expectation that the interaction between MeO and tbpC is ionic. The Lapacian’s positive sign shows that charge is exported from the BCP, and the |2G/V| ratio indicates that the interaction is not covalent. We have already suspected from the short distance between methoxy O and tbpC that the tbC cationic center exerts an electrostatic attraction on the methoxy oxygens. The basin charges for the tbpC (+1.539) and methoxy oxygens (−0.140) are consistent with this inference.

#### 3.4.2. ELF Analysis

**Figure 12:**
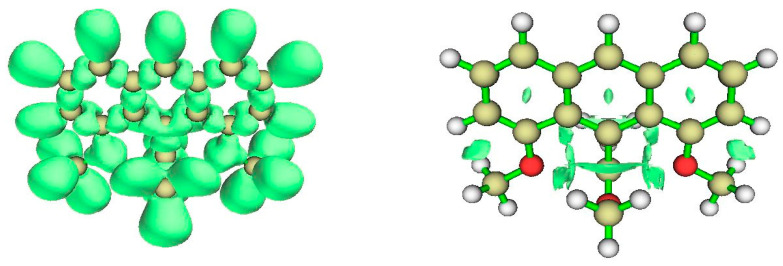
ELF isosurfaces and non-covalent index (NCI) zones for Akiba’s five-coordinated carbon species **4**.

The view of the ELF isosurfaces for Akiba’s five-coordinated C species (Figure 12, left) shows the tbpC-OMe (the clearest view is at lower left) and the OMe connected to the anthryl ring; there is no ELF between that OMe’s O atom and the tbpC. This argues for an interaction dominated by electrostatics.

#### 3.4.3. NCI Analysis

The NCI regions displayed in Figure 12 (right) include the interiors of aromatic rings and the interaction zone between the anthryl methoxy fragment and a nearby CH bond. There is an NCI zone between the OMe attached to the tbpC and the anthryl Carbon to which the tbpC is attached. Most significant, we see an NCI between each anthryl methoxy O atom and the tbpC. There is important interaction, but suggests there is no covalent bonding between the anthryl methoxy O atoms and the tbpC.

## 4. Proposed Symmetric Variants on a Known Example of Approximately Octahedral Coordination of Carbon

Yamaguchi et al. reported the synthesis of a system with six entities in the coordination sphere of a carbon atom [49].

**Figure 13:**
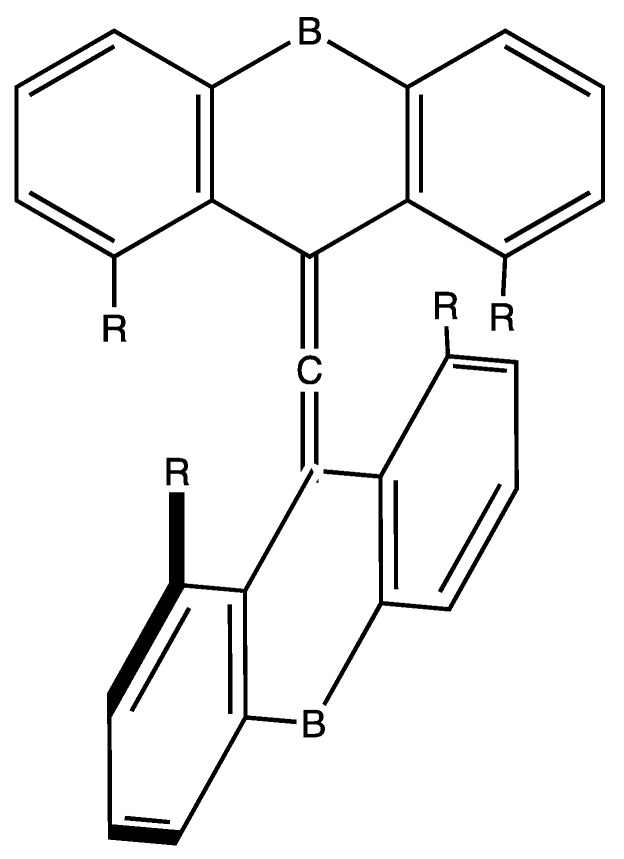
Design for hexacoordinate Carbon (hcC). B is a bridging group, and R is an electron donor.

**Figure 14:**
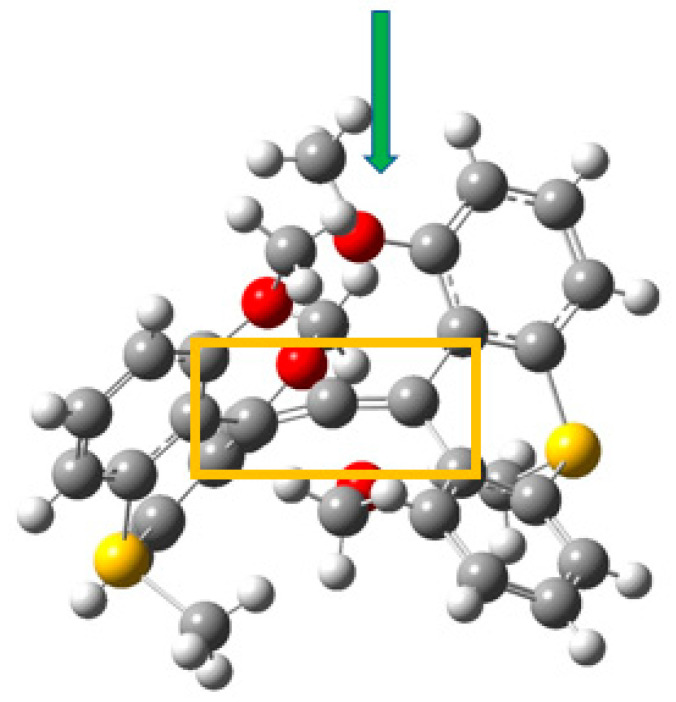
View of Yamaguchi’s allene system with hexacoordinate carbon (species **5**).

The design principle is shown in Figure 13. In Figure 14 showing the X-ray structure of the dication with -SMe(+) bridges, (**5**) the golden rectangle encloses the central six-coordinate carbon (6cC) and its two allenic C neighbors, while the green arrow points to one of the four methoxy oxygen atoms in the coordination sphere of 6cC. The system is far from symmetrical, probably a consequence of packing requirements of the counterions. A variety of related hexacoordinated-C compounds have been explored computationally; for example, in the coordination sphere -OPh can replace -OMe as R and the bridging >SMe (+) can be replaced by >SO_2_, >CH, and >S as B (Figure 13). Substituents can also be chosen which influence the strength of interactions in the coordination sphere [50,51,52].

**Figure 15:**
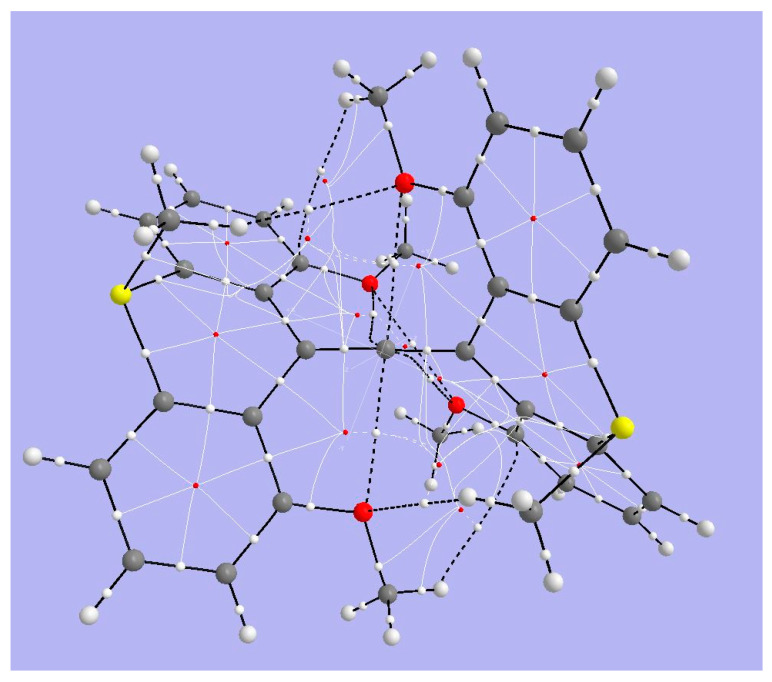
Bader analysis of Yamaguchi’s allene. Large light-gray spheres = H atoms; large dark gray spheres = C atoms; small white spheres = Bond CPs; small red spheres = Ring CPs; solid black lines = bond paths; broken black lines = bond paths for weak interactions.

Since the behavior of this structure is complex, we address only a few details of its topology. Figure 15 displays the results of topological analysis of Yamaguchi’s system, in its crystal geometry. As seen in earlier examples, bond critical points appear on bond paths (heavy black lines) corresponding to links in the molecular drawing. Ring critical points (in red) are found within each of the aromatic rings of the anthryl fragments. Fine white lines connect RCPs and BCPs. Heavy broken lines represent weaker interactions. The four methoxy oxygens are linked by such lines to the central hexacoordinate C. We also observe heavy broken lines connecting the methoxy oxygen atoms with H atoms of the thiomethyl bridging groups, among other surprising connections.

We may expect that the Bader parameters for the X-ray structure, obtained by analysis of a charge distribution that does not correspond to an energy minimum within that computational method, might be called into question. We should judge the results by their consistency with more rigorous calculations on similar systems, as described below. The density and Laplacian values at the BCPs for the allene fragment are conventional for double bonds, and the interaction between methoxy O atoms and the hcC is similar to that found in Akiba’s system for its methoxy O atoms and the pcC. (Table 4 and Table 5). That is, the BCPs for the bond path between MeO and the hcC have small positive ∇^2^*ρ* values and |2G/V| ratios > 1. This indicates charge depletion at the BCP and predominantly e electrostatic interactions. Since the AIM charges of these O atoms and the hcC are both negative, we can confirm that the electrostatic interaction is repulsive contrary to the suggestion that the interaction is stabilizing by mixing of the methoxy O lone pair with the allyl π* MO and the -OMe fragments are forced into the hcC coordination sphere solely by steric constraint.

Owing to the difficulty of visualization in this asymmetric system, we do not discuss the ELF and NCI figures. Instead we turn to discussion of our proposed symmetric variants.

### 4.1. A Neutral Symmetric System with Six-Coordinate Carbon

**Figure 16:**
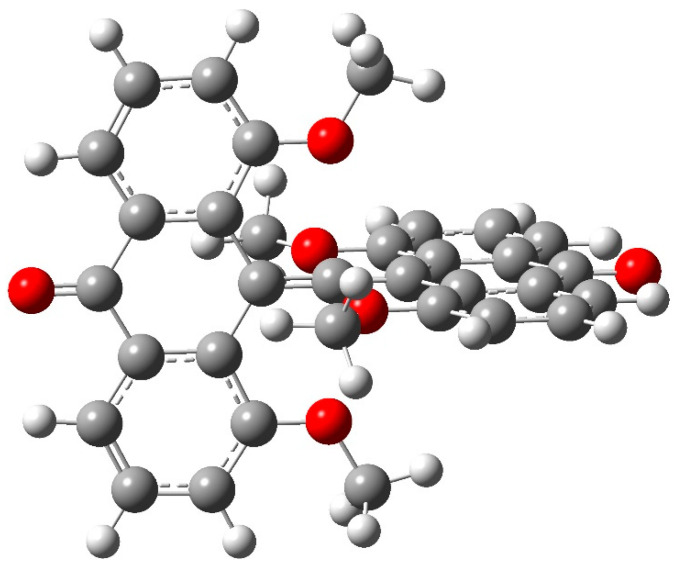
D_2d_ neutral symmetric variant with >C=O bridge, species **6**.

In the example **6** shown in Figure 16 the bridge is >C=O, replacing the >SMe(+) of the first experimental compound **5**. High symmetry allows a clearer view of the geometry of our variants. We wish to encourage synthesis of this neutral system and its -SCH_3_ analog described below. A neutral charge allows for the possibility of high symmetry in the solid phase, thereby simplifying the X-ray analysis.

#### 4.1.1. AIM Analysis

**Figure 17:**
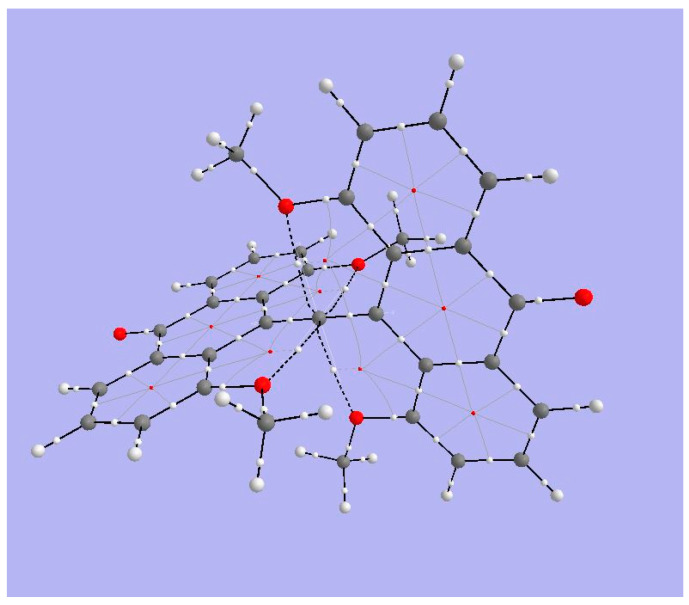
AIM analysis of CO system **6**.

Symmetry equivalence also allows a compact presentation of AIM results, shown in Figure 17 for the >C=O system **6**. Some key numerical values for density, the Laplacian, and energy densities along with the ratio |2G/V| are collected in Table 6. The parameters reflect the double bond character of the allenic core containing the hcC and the polar nature of the H_3_C-O and C=O bonds. The small values of density and the Laplacian *L*, and the positive sign of *L* as well, attest to the weak and predominantly electrostatic nature of the interaction.

#### 4.1.2. ELF Analysis

**Figure 18:**
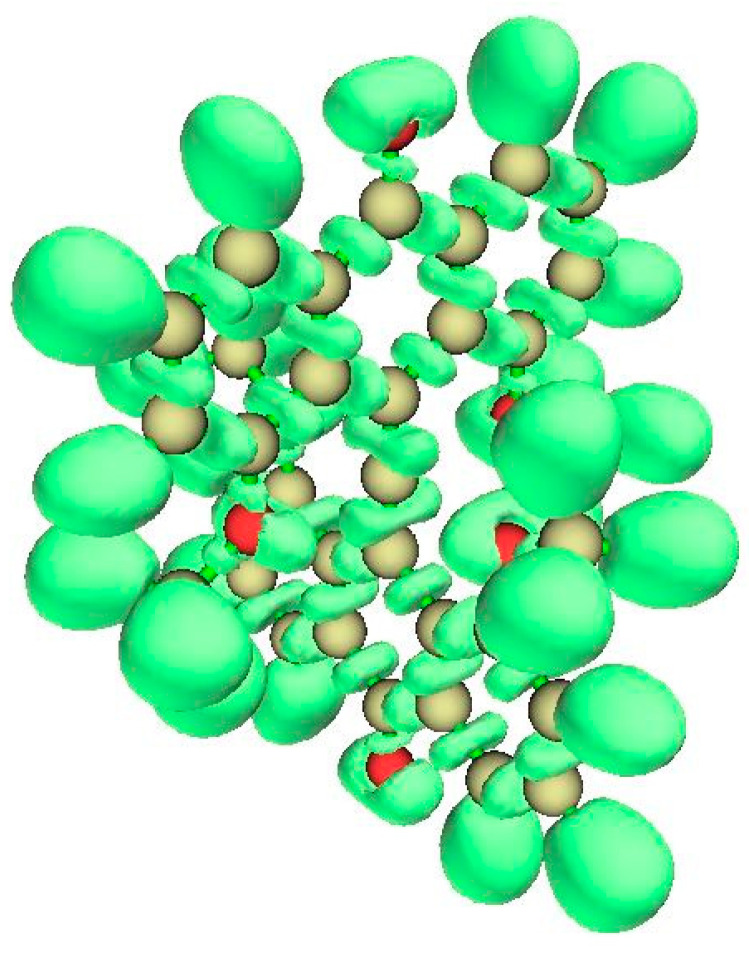
ELF for the D_2d_ (>CO bridge) variant of Yamaguchi’s system.

The electron localization function (Figure 18) shows the central allene system, with the anthryl methoxy oxygens in the coordination sphere of the hexacoordinated C. There is no indication of ELF amplitude in the bonding region between CH_3_O and the hcC, but rather an accumulation of charge on the methoxy oxygen and depletion of charge near the hcC. This is entirely consistent with the description of the interaction between MeO and hcC as weak and electrostatic, inferred from the AIM analysis.

#### 4.1.3. NCI Analysis

**Figure 19:**
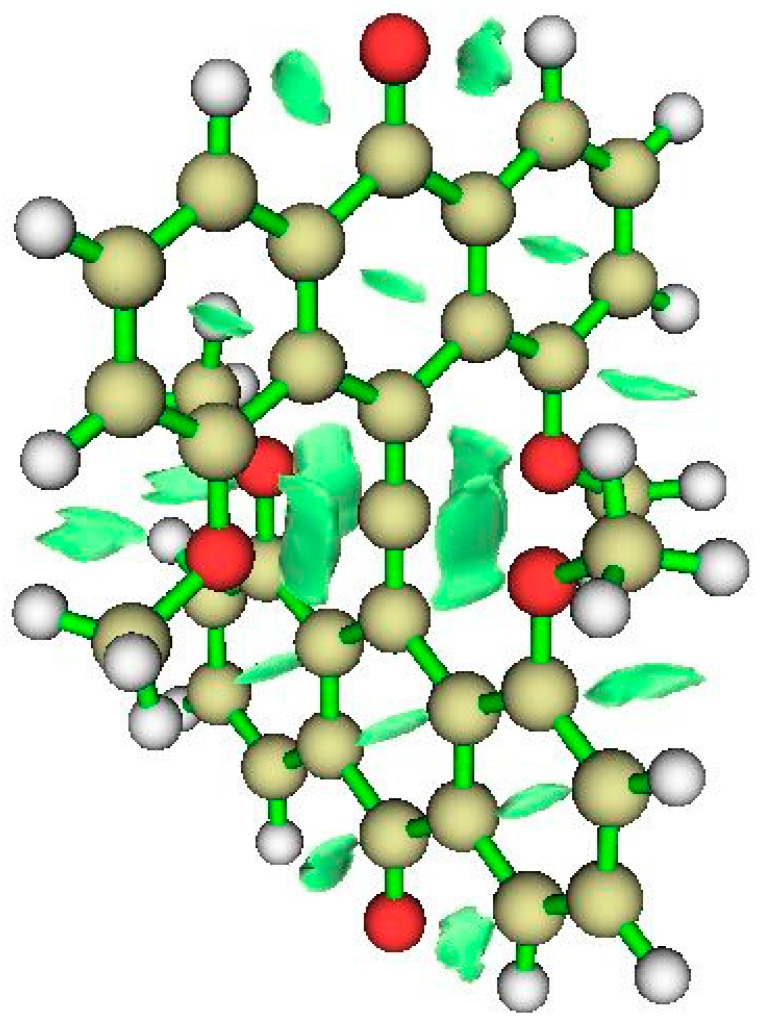
NCI for CO neutral variant.

The NCI zones displayed in Figure 19 include interactions between the bridging >C=O groups and ipso CH groups of the anthryl fragments, destabilized regions within six-membered rings, and interactions between -OCH_3_ methyl groups and nearby CH groups of the anthryl frame. Most significant for our purpose are noncovalent interactions between -OCH_3_ and the allenyl group’s central hexacoordinated carbon.

### 4.2. A Neutral System with Attractive Interactions in the Coordination Sphere

**Figure 20:**
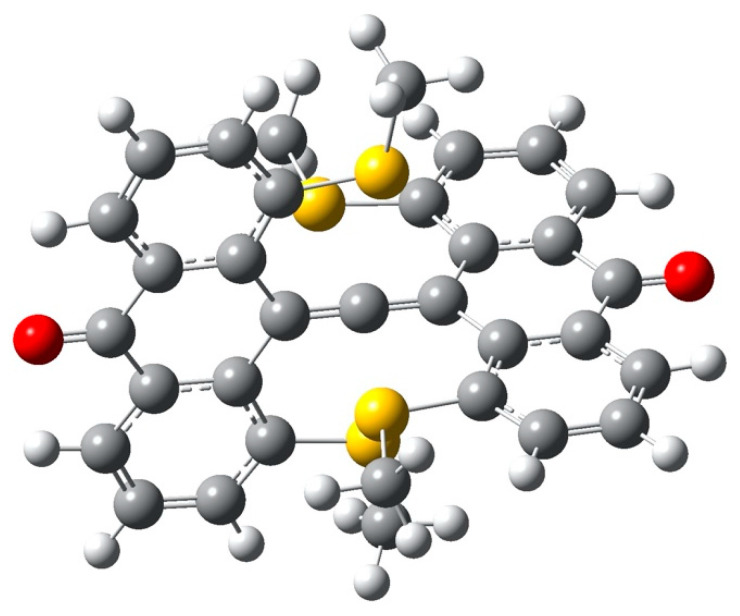
Structure of carbonyl-bridged system with -SCH_3_ (**7**).

To enhance possible soft–soft interaction between the six-coordinate Carbon and members of its coordination set we replace the methoxy O atoms by Sulfur atom, retaining D_2d_ symmetry. The structure **7** is shown in Figure 20.

#### 4.2.1. AIM Analysis

**Figure 21:**
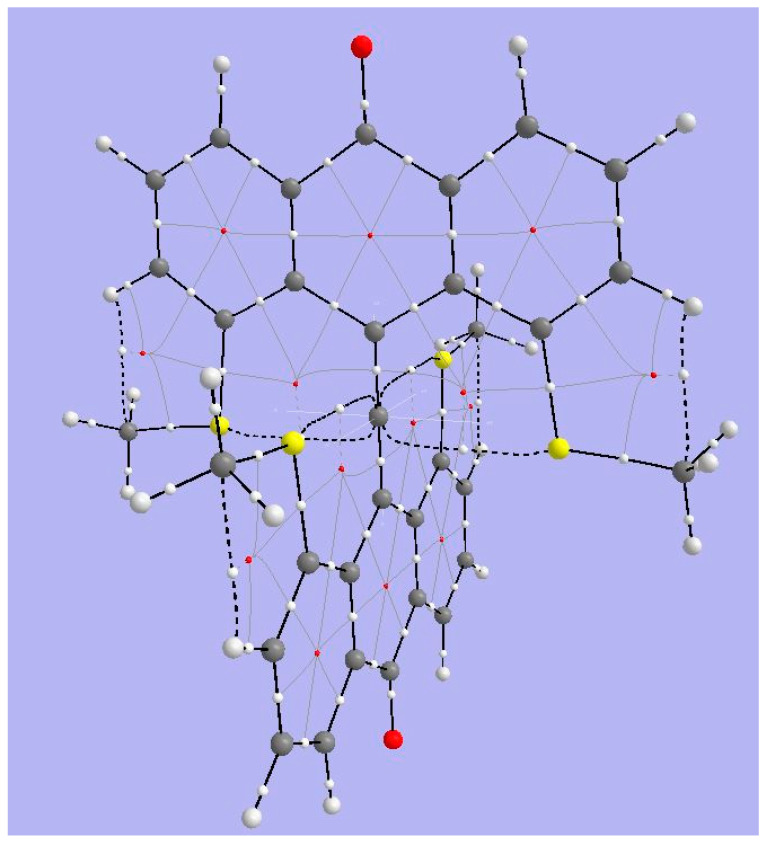
D_2d_ carbonyl bridge thiamethyl variant.

Figure 21 displays the familiar array of bond critical points (CH, CC, C=O, CS, including ring CPs for each six-membered ring. The S…hcC bond paths are curved oddly in the neighborhood of the hcC, and there is an unexpected weak bond from anthryl CH hydrogen to S-CH_3_ methyl carbon.

According to the AIM data (Table 7), the strength of the CH_3_S…C (central) interaction is almost as strong as the CH_3_O…C (central) interaction. Compare the Laplacian value of 0.0619 for **7** with +0.0755 for **6**.

As was the case for the methoxy system’s O…hcC BCP, the S…hcC BCP has a positive Laplacian, indicating charge depletion at that point, and predominantly electrostatic interaction. But in this case the interaction is attractive; hcC is negatively charged in each case, but the CH_3_S sulfur atom has a positive AIM charge whereas the CH_3_O oxygen atom has a strongly negative AIM charge. (Table 8) This qualitative difference is unaffected by enhancing the basis from cc-pVTZ to aug-cc-pVTZ, though the charges in the thia system are appreciably altered.

#### 4.2.2. ELF Analysis

**Figure 22:**
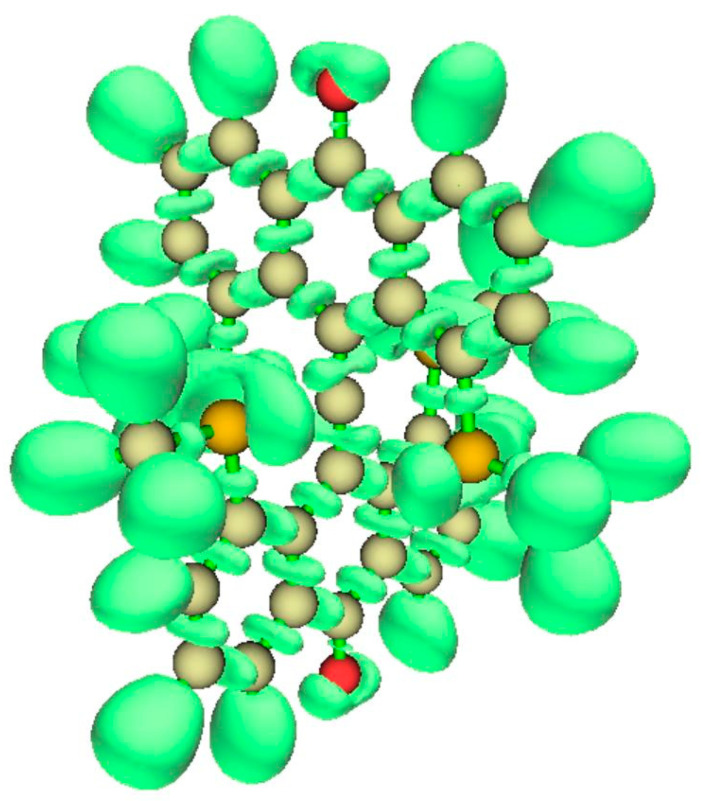
ELF visualization for thia variant. Oxygen atoms are shown in red, sulfur atoms in brown, and carbon atoms in gray.

In Figure 22, the ELF is again shown as a set of isosurfaces enclosing regions of high charge localization. Along the axis linking the carbonyl oxygens and passing through the allenyl carbons we see features corresponding to the carbonyl oxygen lone pairs (top and bottom) and also the orthogonal pi bonds between the central hcC and its C neighbors in the allene. Two of the four S atoms (from anthryl -SMe substituents) are in view, at right and left. Lone pairs on S atoms are prominent, but there is no evident localization between S atoms and the hcC. This is consistent with the low value of electron density at the BCP on the path fromS to hcC, and confirms the essentially electrostatic nature of the interaction.

#### 4.2.3. NCI Analysis

**Figure 23:**
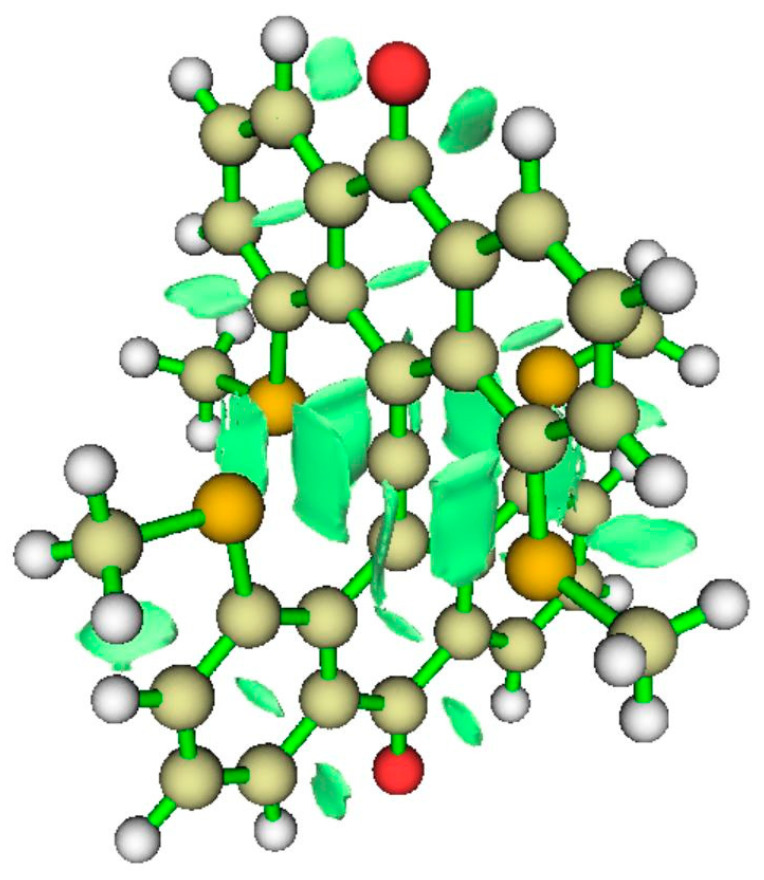
NCI for the thia variant.

In Figure 23, we find regions of non-covalent interaction (a) between anthryl carbonyl groups and nearby anthryl CH hydrogen atoms; (b) between -SCH_3_ methyls and nearby anthryl CH hydrogens; and (c) within anthryl rings. Most prominent are the NCI regions between S atoms and the central hcC.

## 5. Conclusions

We examined some well-established examples of unusual coordination of carbon atom. Systems **1** and **3** containing tetracoordinate planar carbon (tpC) are stabilized by removal of two electrons; in Radom’s octaplanes the tpCC_4_^2+^ fragment is further constrained by surrounding hydrocarbon rings. To our knowledge no X-ray structures are known for these systems, though many analogs with metal substituents have been characterized. [17,18,19,20,21] We recommend that the synthesis and spectroscopic characterization of hydrocarbon octaplanes be re-visited.

A signal experimental success is the structural study of hexamethyl benzene dication containing hexacoordinated carbon at the apex of a pentagonal pyramid. Bond paths confirm hexacoordination, but need not correspond to electron pair bonds. This electron-deficient species, like many carbocations with unusual carbon coordination, does not threaten the octet rule.

Building on a theme first illustrated in a cationic 1,8-dimethoxy-9-dimethoxymethylanthracene, a series of five- and six-coordinated species have been synthesized [46,47,48,49,50]. In the pentacoordinate system two of the O atoms in the coordination sphere are bound weakly. AIM, ELF, and NCI analysis all confirm that for an experimental system possessing six entities in a C atom’s coordination sphere, and for symmetrized variations proposed here, there is evidence for six significant interactions between the allenyl central C and neighbors. The four interactions with O (**6**) or S (**7**) are electrostatic, not covalent, in view of the low level of charge sharing and density at the bond critical points. The C is hexacoordinate but not hexavalent. Again, no violation of the octet rule model is claimed.

Replacing the oxygen in the methoxy group with sulfur alters the electrostatic interaction from repulsive to attractive. We encourage synthesis of species **6** and **7**, and a thorough structural characterization including X-ray, vibrational, and NMR spectra. This should distinguish the repulsive character in **6** from the attractive character of **7**.

## Figures and Tables

**Table 1:** Selected AIM parameters for spiropentadiene dication. Cx is a peripheral Carbon, tetra-coordinate carbon as in planar methane (tpC) is the tetracoordinate planar Carbon. Red: From Firme et al. [41].

Critical Point	Density *ρ*	Laplacian	G	V	|2G/V|
tpC-Cx: (3, −1)	0.2323 0.230	−0.1184 −0.113	+0.1685	−0.3506	0.961
Cx-Cx (3, −1)	0.3708 0.369	−1.1167 −1.109	+0.1872	−0.6535	0.573
Cx-H (3, −1)	0.2716 0.275	−1.0302 −1.050	+0.0134	−0.2844	0.094
Basin Charges					
Charge (|e|)	H = +0.3794	Cx = +0.1691		tpC = −0.1932	

**Table 2:** Selected Atoms in Molecules (AIM) parameters for hexamethylbenzene dication.

Critical Point	Density	Laplacian	G	V	|2G/V|
C ring-C apex: (3, −1)	0.1551	+0.0205	+0.0913	−0.1765	1.0346
C ring-C ring (3, −1)	0.2823	−0.7869	+0.0894	−0.3766	0.4748
C apex-Me (3, −1)	0.2705	−0.5607	+0.0962	−0.3254	0.5913
C ring-Me (3, −1)	0.2722	−0.6231	+0.0819	−0.3194	0.5128
Basin Charges					
Charge (|e|)	hcC −0.088	Ring C −0.019	Apical methyl C −0.016	Ring methyl C −0.037	

**Table 3:** Selected Bader parameters for Radom’s dimethanospiro[2.2]octaplane dication **3.**

Critical Point	Density	Laplacian	G	V	|−2G/V|
rC–tcC: (3, −1)	0.2286	−0.2823	+0.1435	−0.3576	0.8026
rC–rC (3, −1)	0.2981	−0.8509	+0.1084	−0.4295	0.5048
sC–sC (3, −1)	0.2493	−0.6234	+0.0729	−0.3016	0.4834

**Table 4:** AIM features for 5C carbon; red entries from Akiba et al. obtained with the B3LYP/6-31G(d) model.

Critical Point	Density	Laplacian	G	V	|2G/V|
tbpC-OMe: (3, −1)	0.3537	−0.3326	+0.4764	−1.0359	0.9198
tbpC…OMe (3, −1)	0.0235 0.022	+0.0898 +0.078	+0.0209	−0.0193	2.1658
C-O (3, −1)	0.2400	−0.6625	+0.2650	−0.5445	0.9734
C=C (3, −1)	0.2618	−1.0337	+0.0708	−0.3073	0.4608

**Table 5:** Bader parameters for the experimental structure of Yamaguchi’s hexacoordinate (hcC) system.

Critical Point		Density *ρ*	Laplacian ∇^2^*ρ*	Kinetic Energy Density G	Potential Energy Density V		|2G/V|
Allenic hcC=Ca		+0.3444	−1.0187	+0.1551	−0.5648		0.5492
Allenic hcC=Cb		+0.3385	−0.9761	+0.1515	−0.5470		0.5539
CH3Oa…hcC		+0.0182	+0.0584	+0.0128	−0.0105		2.4381
CH3Ob…hcC		+0.0192	+0.0739	+0.0165	−0.0146		2.2603
CH3Oc…hcC		+0.0140	+0.0593	+0.0155	−0.0136		2.2794
CH3Od…hcC		+0.0133	+0.0602	+0.0123	−0.0099		2.4848
Basin Charges							
Atom	hcC	Allene Ca	Allene Cb	CH3Oa	CH3Ob	CH3Oc	CH3Od
AIM Q	−0.1943	+0.0926	+0.0898	−1.0736	−1.0884	−1.0753	−1.0720

**Table 6:** Selected Bader parameters for D_2d_ variant (**6**) with >C=O bridging.

Critical Point	*ρ*	Laplacian	G	V	|2G/V|
C=O bond (3, −1)	0.3976	+0.1874	+0.7174	−1.3880	1.0337
C=C bond (3, −1)	0.3455	−1.0337	+0.1576	−0.5736	0.5495
H_3_C-O bond (3, −1)	0.2523	−0.2481	+0.3985	−0.6692	1.1910
MeO … C (3, −1)	0.0203	+0.0755	0.0171	−0.0154	2.2207

**Table 7:** Selected Bader parameters for D_2d_ thia methyl variant (**7**) with >C=O bridging.

Critical Point	*ρ*	Laplacian	G	V	|2G/V|
C=O bond (3, −1)	0.4045	−0.0281	+0.6700	−1.3470	0.9948
C=C bond (3, −1)	0.3448	−1.0315	+0.1624	−0.5826	0.5575
H_3_C-S bond (3, −1)	0.1845	−0.2965	+0.0497	−0.1736	0.5726
MeS … C (3, −1)	0.0201	+0.0619	+0.0141	−0.0128	2.3125

**Table 8:** AIM Charges for atoms in the hcC coordination zone for species **6** and **7**.

Basis cc-pVTZ			
Charges (O)	C(allyl)	hcC	O atom
Species **6**	+0.2193	−0.3279	−1.1729
Charges (S)	C(allyl)	hcC	S atom
Species **7**	+0.1552	−0.3484	+0.1130
Basis aug-cc-pVTZ			
Charges (O)	C(allyl)	hcC	O atom
Species **6**	+0.2213	−0.3256	−1.1815
Charges (S)	C(allyl)	hcC	S atom
Species **7**	+0.2077	−0.4591	+0.1087

## Supplementary Materials

The following are available online. Analysis of the D_2d_ > CF(+) bridged hcC species, Plots of RDG vs λ_2_-signed density for Structures **1**–**4**. Analysis of the D_2d_ > SO_2_ bridged species. Cartesian coordinates for species **1**–**4**, **6**, and **7**.

## References

[1] Hoff V.J.H. (1874). Sur les formules de structure dans l’espace. Arch. Néerl. Sci. Exact. Nat..

[2] Le Bel J.A. (1874). Sur les relations qui existent entre les formules atomiques des corps organiques et le pouvoir rotatoire de leurs dissolutions. Bull. Soc. Chim. Fr..

[3] Leicester H.M., Gillispie C.C. (1973). Le Bel, Joseph.Achille. Dictionary of Scientific Biography.

[4] Snelders H.A.M., Gillispie C.C. (1976). van’t Hoff, Jacobus Hendricus. Dictionary of Scientific Biography.

[5] Gillespie R.J., Nyholm R.S. (1957). Inorganic Stereochemistry. Quart. Rev. Chem. Soc..

[6] Gillespie R.J. (2008). Fifty years of the VSEPR model. Coord. Chem. Rev..

[7] Hoffmann R., Alder R.W., Wilcox C.F. (1970). Planar Tetracoordinate Carbon. J. Am. Chem. Soc..

[8] Hoffmann R. (1970). The Theoretical Design of Novel Stabilized Systems. Pure. Appl. Chem..

[9] Wong M.W., Radom L. (1989). Methane Dication: Planar but Not Square. J. Am. Chem. Soc..

[10] Lammertsma K., Schleyer P.v.R. (1988). Structures and energies of isomeric carbodications (C_5_H_4_^2+^ and C_6_H_4_^2+^). J. Phys. Chem..

[11] Hogeveen H., Kwant P.W. (1973). Direct observation of a remarkably stable dication of unusual structure: (CCH_3_)_6_^2+^. Tetrahedron Lett..

[12] Hogeveen H., Kwant P.W., Postma J., van Duynen P.T. (1974). Electronic spectra of pyramidal dications, (CCH_3_)_6_^2+^ and (CH)_6_^2+^. Tetrahedron Lett..

[13] Hogeveen H., Kwant P.W. (1974). Chemistry and spectroscopy in strongly acidic solutions XL. (CCH_3_)_6_
^2+^ an unusual dication. J. Am. Chem. Soc..

[14] Radom L., Rasmussen D.R. (1998). The planar carbon story. Pure Appl. Chem..

[15] Lyons J.E., Rasmussen D.R., McGrath M.P., Nobes R.H., Radom L. (1994). Octaplane: A Saturated Hydrocarbon with a Remarkably Low Ionization Energy Leading to a Cation with a Planar Tetracoordinate Carbon Atom. Angew. Chem. Int. Ed. Engl..

[16] Wang Z.X., Von Ragué Schleyer P. (2002). The Theoretical Design of Neutral Planar Tetracoordinate Carbon Molecules with C(C)_4_ Substructures. J. Am. Chem. Soc..

[17] Von Ragué Schleyer P., Boldyrev A.I. (1991). A new, general strategy for achieving planar tetracoordinate geometries for carbon and other second row periodic elements. J. Chem. Soc. Chem. Comm..

[18] Li X., Wang L.S., Boldyrev A.I., Simons J. (1999). Tetracoordinated planar carbon in the Al_4_C-anion. A combined photoelectron spectroscopy and ab initio study. J. Am. Chem. Soc..

[19] Boldyrev A.I., Simons J. (1998). Tetracoordinated Planar Carbon in Pentaatomic Molecules. J. Am. Chem. Soc..

[20] Keese R. (2006). Carbon Flatland: Planar Tetracoordinate Carbon and Fenestranes. Chem. Rev..

[21] Wu X.-F., Cheng Y.-X., Guo J.-C. (2019). CLi_2_AlE (E = P, As, Sb, Bi): Planar Tetracoordinate Carbon Clusters with 16 and 14 Valence Electrons. ACS Omega.

[22] Exner K., Schleyer P.v.R. (2000). Planar Hexacoordinate Carbon: A Viable Possibility. Science.

[23] Bader R.F.W. (1994). Atoms in Molecules: A Quantum Theory.

[24] Becke A.D., Edgecombe K.E. (1990). A simple measure of electron localization in atomic and molecular systems. J. Chem. Phys..

[25] Johnson E.R., Keinan S., Mori-Sánchez P., Contreras-García J., Cohen A.J., Yang W. (2010). Revealing noncovalent interactions. J. Am. Chem. Soc..

[26] Frisch M.J., Trucks G.W., Schlegel H.B., Scuseria G.E., Robb M.A., Cheeseman J.R., Scalmani G., Barone V., Petersson G.A., Nakatsuji H. (2016). Gaussian 09, Revision A.02.

[27] Frisch M.J., Trucks G.W., Schlegel H.B., Scuseria G.E., Robb M.A., Cheeseman J.R., Scalmani G., Barone V., Petersson G.A., Nakatsuji H. (2016). Gaussian 16, Revision C.01.

[28] Chai J.-D., Head-Gordon M. (2008). Long-range corrected hybrid density functionals with damped dispersion corrections. Phys. Chem. Chem. Phys..

[29] Dunning T.H. (1989). Gaussian basis sets for use in correlated molecular calculations. I. The atoms boron through neon and hydrogen. J. Chem. Phys..

[30] Todd A., Keith T.K. (2017). AIMAll, Version 17.11.14.

[31] Lu T., Chen F. (2012). Multiwfn: A Multifunctional Wavefunction Analyzer. J. Comp. Chem..

[32] Contreras-García J., Johnson E.R., Keinan S., Chaudret R., Piquemal J.P., Beretan D., Yang W. (2011). NCIPLOT: A program for plotting noncovalent interaction regions. J. Chem. Theory Comp..

[33] Kumar P.S.V., Raghavendra V., Subramanian V.J. (2016). Bader’s theory of atoms in molecules (AIM) and its applications to chemical bonding. J. Chem. Sci..

[34] Grabowski S.J. (2011). What is the Covalency of Hydrogen Bonding?. Chem. Rev..

[35] Grimme S., Mueck-Lichtenfeld C., Erker G., Kehr G., Wang H., Beckers H., Willner H. (2009). When do interacting atoms form a chemical bond? Spectroscopic measurements and theoretical analyses of dideuteriophenanthrene. Angew. Chem. Int. Ed..

[36] Bader R.F.W. (2009). Bond Paths are Not Chemical Bonds. J. Phys. Chem. A.

[37] Cerpa E., Andreas Krapp A., Flores-Moreno R. (2009). Influence of Endohedral Confinement on the Electronic Interaction between He atoms: A He2@C20H20 Case Study. Chem. Eur. J..

[38] Clark T., Murray J.S., Politzer P. (2018). A perspective on quantum mechanics and chemical concepts in describing noncovalent interactions. Phys. Chem. Chem. Phys..

[39] Narth C., Maroun Z., Boto C.R., Bonnet M.-L., Piquemal J.-P., Contreras-Garcia J., Alikhani E., Chauvin R., Lepetit C., Silvi B. (2016). A Complete NCI Perspective from new bonds to reactivity. Applications of Topological Methods in Molecular Chemistry.

[40] Esteves P.M., Ferreira N.B.P., Corrêa R.J. (2005). Neutral Structures with a Planar Tetracoordinated Carbon Based on Spiropentadiene Analogues. J. Am. Chem. Soc..

[41] Firme C.L., Barreiro N.B.P., Esteves P.M., Corrêa R.J. (2008). Understanding the Planar Tetracoordinate Carbon Atom: Spiropentadiene Dication. J. Phys. Chem. A.

[42] Firme C.L., Antunes O.A.C., Esteves P.M., Corrêa R.J. (2009). Derivatives of Spiropentadiene Dication: New Species with Planar Tetracoordinate Carbon (ptC) atom. J. Phys. Chem. A.

[43] Malischewski M., Seppelt K. (2017). Crystal Structure Determination of the Pentagonal-Pyramidal Hexamethylbenzene Dication (CH_3_)_6_C_6_^2+^. Angew. Chem. Int. Ed..

[44] Schleyer P.v.R., Maerker C., Dransfeld A., Jiao H., van Eikema Hommes N.J. (1996). Nucleus-Independent Chemical Shifts: A Simple and Efficient Aromaticity Probe. J. Am. Chem. Soc..

[45] Klein J.E.M.N., Havenith R.W.A., Knizia G. (2018). The Pentagonal-Pyramidal Hexamethylbenzene Dication: Many Shades of Coordination Chemistry at Carbon. Chem. Eur. J..

[46] Akiba K., Yamashita M., Yamamoto Y., Nagase S. (1999). Synthesis and Isolation of Stable Hypervalent Carbon Compound (10-C-5) Bearing a 1,8-Dimethoxyanthracene Ligand. J. Am. Chem. Soc..

[47] Yamamoto Y., Akiba K. (2004). Synthesis of Hypervalent Pentavalent Carbon and Boron Compounds. J. Synth. Org. Chem. Jpn..

[48] Yamashita M., Yamamoto Y., Akiba K., Hashizume D., Iwasaki F., Tagaki N., Nagase S. (2005). Synthesis and Structures of Hypervalent Carbon and Boron Compounds Bearing an Anthracene Skeleton–Elucidation of Hypervalent Interaction Based on X-ray Analysis and DFT Calculation. J. Am. Chem. Soc..

[49] Yamaguchi T., Yamamoto Y., Kinoshita D., Akiba K., Zhang Y., Reed C.A., Hashizune D., Iwasaki F. (2008). Synthesis and Structure of a Hexacoordinate Carbon Compound. J. Amer. Chem. Soc..

[50] Yamaguchi T., Yamamoto Y. (2013). Substituent effects on the structure of hexacoordinate carbon bearing two thioxanthene ligands. Pure Appl. Chem..

[51] Nakai H., Okoshi M., Atsumi T., Kikuchi T., Akiba K. (2011). Theoretical Design of Hexacoordinate Hypervalent Carbon Compounds by Analyzing Substituent Effects. Bull. Chem. Soc. Jpn..

[52] Kikuchi Y., Ishii M., Akiba K., Nakai H. (2008). Discovery of hexacoordinate hypervalent carbon compounds: Density functional study. Chem. Phys. Lett..

