# Supplementary material for: Bonding Analysis of Compounds with Unusual Coordination of Carbon: Proposed Symmetric Systems with Six-Coordinate Carbon
Source: Molecules. 2020 Aug 28;25(17):3937. doi: 10.3390/molecules25173937 (PMC7504323; doi:10.3390/molecules25173937)
Supplement: Supplementary file 1 [file molecules-25-03937-s001.pdf]

molecules

MDPI

Bonding Analysis of Six-Coordinate Carbon in Allene Systems

Zikri Altun, Physics Department, Marmara University, Istanbul, TURKEY
Carl Trindle, Chemistry Department of Chemistry, University of Virginia, USA

Table of Contents

SUPPLEMENTAL INFORMATION 2

DICATIONIC SYMMETRIC SYSTEMS: CF(+) BRIDGE (S1) 2
AIM ANALYSIS 3
ELF ANALYSIS 6
NCI ANALYSIS 6
D_{2H} SPIROPENTADIENE DICATION: PLOT OF RDG VS λ2-SIGNED DENSITY 8
HEXAMETHYL BENZENE DICATION: PLOT OF RDG VS λ2-SIGNED DENSITY 8
RADOM'S DIMETHANOSPIRO[2.2]OCTAPLANE DICATION 9
YAMAGUCHI'S FIVE-COORDINATE CARBON COMPOUND: PLOT OF RDG VS λ2-SIGNED DENSITY 9
FURTHER DEPICTION OF THE (–SME, >C=O) SYSTEM 10
NEUTRAL SYMMETRIC SYSTEMS: >SO_2 BRIDGE (S2) 15
AIM ANALYSIS 16
ELF ANALYSIS 17
NCI ANALYSIS 18
RESPONSE TO ENHANCED BASIS SET 19
COORDINATES FOR SYSTEMS STUDIED 20
SPIROPENTADIENE DICATION (**1**) 20
HEXAMETHYL BENZENE DICATION (**2**) 20
DIMETHANOSPIRO[2.2]OCTAPLANE DICATION (**3**) 21
1,8-DIMETHOXY-9-DIMETHOXY ANTHRACENE CATION (**4**) 22
NEUTRAL CO BRIDGED -OCH_3 VARIANT OF YAMAGUCHI'S HEXACOORDINATED CARBON COMPOUND (**6**) 23
NEUTRAL CO BRIDGED -SCH_3 VARIANT OF YAMAGUCHI'S HEXACOORDINATED CARBON COMPOUND (**7**) 25
DICATIONIC CF BRIDGED -OCH_3 VARIANT OF YAMAGUCHI'S HEXACOORDINATED CARBON COMPOUND (**S1**) 26
NEUTRAL SO_2 BRIDGED -OCH_3 VARIANT OF YAMAGUCHI'S HEXACOORDINATED CARBON COMPOUND (**S2**) 28

Figure 1: View of the six-coordinated system with >CF(+) replacing >SMe 3
Figure 2: Topological sketch for a symmetrized variant of Yamaguchi's six-coordinate system 4
Figure 3: Electron localization function for the D2 variant with the >CF(+) bridge 6
Figure 4: Identifier of zones with small signed density and small RDG for the CF(+) bridged D2d variant 7

Figure 5: NCI for CF(+) variant 7
Figure 6: RDG vs sign(λ2)ρ for spiropentadiene dication 8
Figure 7:RDG vs λ2-signed density shows two non-covalent interactions 8
Figure 8: RDG vs λ2-signed density for Radom's dimethanospiro[2.2]octaplane dication shows a number of weak interactions 9
Figure 9: RDG vs λ2-signed density for 1,8-dimethoxy-9-dimethoxymethylanthracene cation (top) and its thia-variant (bottom) show a number of weak interactions 10
Figure 10: NCI contour plot for D_{2d} species 10
Figure 11: ELF function along the line connecting the allene carbons 11
Figure 12: ELF function along the line S through allene to S 12
Figure 13: ELF on the line from S to the allene central carbon 12
Figure 14: Plot RDG vs λ2-signed density for the thia variant of show a number of weak interactions 13
Figure 15: Contour diagram of NCI in one anthryl plane 14
Figure 16: Structure of the >SO_2 bridged variant of Yamaguchi's hcC system 15
Figure 17: AIM critical points and bond paths for >SO2 bridged variant of Yamaguchi's hcC system 16
Figure 18: ELF isosurface for the >SO2 bridged variant of Yamaguchi's hcC compound 17
Figure 19: NCI zones for the >SO_2 – bridged variant of Yamaguchi's hcC system 18

Table 1: Selected Bader parameters for the CF D_{2d} variant 5
Table 2: Bader parameters for the >SO2 - bridged neutral variant of Yamaguchi's hcC compound 17
Table 3: Selected Bader parameters for D2d variant (6) with >C=O bridging (cc-pVTZ) 19
Table 4: Selected Bader parameters for D2d variant (6) with >C=O bridging (aug-cc-pVTZ) 19
Table 5: AIM Charges for atoms in the hcC coordination zone for species 6 and 7. (cc-pVTZ) 19
Table 6: AIM Charges for atoms in the hcC coordination zone for species 6 and 7. (aug-cc-pVTZ) 19
Table 7: AIM Charges for atoms in the hcC coordination zone for species 6 and 7. (aug-cc-pVTZ) 19
Table 8: Selected Bader parameters for D2d thia methyl variant (7) with >C=O bridging (aug-cc-pVTZ) 20

Supplemental Information

Dicationic symmetric systems: CF(+) bridge (**S1**)

Figure 1 shows the CF(+)-bridged variant of Yamaguchi's hexacoordinate Carbon compound. The >CF(+) bridged system attains D_{2d} symmetry in the absence of counter-ions. The neutral >C=O bridged systems discussed in the primary report prefer D_{2d} symmetry, which may be preserved in the condensed phase.

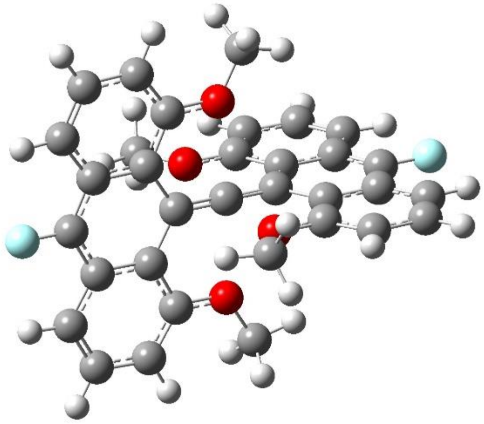

Figure 1: View of the six-coordinated system with >CF(+) replacing >SMe

The D_{2d} axis passes through the F atoms. The symmetry simplifies the tabulation of bond critical points, discussed below.

AIM analysis

The topological diagram shown in Figure 2 includes conventional BCPs, RCPs in the anthryl rings, and (in broken lines connecting four methoxy O atoms with the hcC) the bond paths completing the six objects in its coordination sphere.

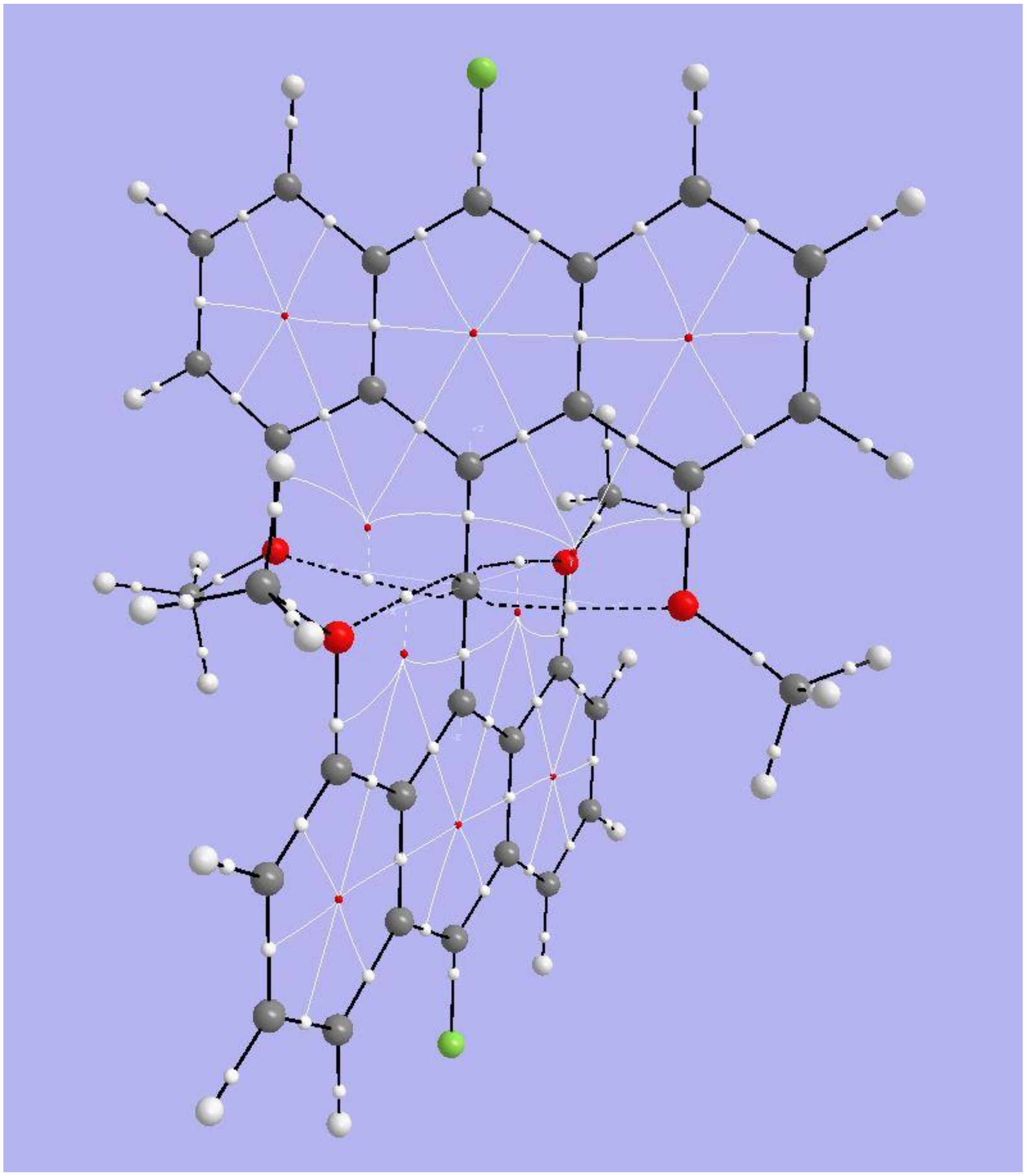

Figure 2: Topological sketch for a symmetrized variant of Yamaguchi's six-coordinate system

In Table 1 we list only the BCPs for CF, the allyl C=C bond, the Methoxy C-O bond, besides the CP on the path from the Methoxy O to the hcC. Selected AIM basin charges are reported below.

Critical Point	ρ	Laplacian	G	V	|2G/V|
C=O bond (3,-1)	0.3976	+0.1874	+0.7174	-1.3880	1.0337
C=C bond (3,-1)	0.3455	-1.0337	+0.1576	-0.5736	0.5495

H_3C-O bond (3,-1)	0.2523	-0.2481	+0.3985	-0.6692	1.1910
MeO … C (3,-1)	0.0203	+0.0755	0.0171	-0.0154	2.2207

Charges (absolute electrons) on selected atomic basis are listed below

hcC	Allene C	Coordinated O	Bridging C	F on bridging C
-0.3002	+0.2473	-1.1719	+0.6140	-0.6549

Table 1: Selected Bader parameters for the >CF(+) D_{2d} variant

ELF analysis

The ELF isosurface graph (Figure 3) indicates the substantially ionic bond CF, the pi bonds of the central allyl fragment, and the bonds from each of the terminal carbons of the allyl fragment linking to the anthryl segment. A key feature is the ELF distribution between the methoxy O and the hcC; the ELF lobe corresponds to an O lone pair, and there is no substantial ELF shared with the hcC. This already indicates that the interaction is ionic.

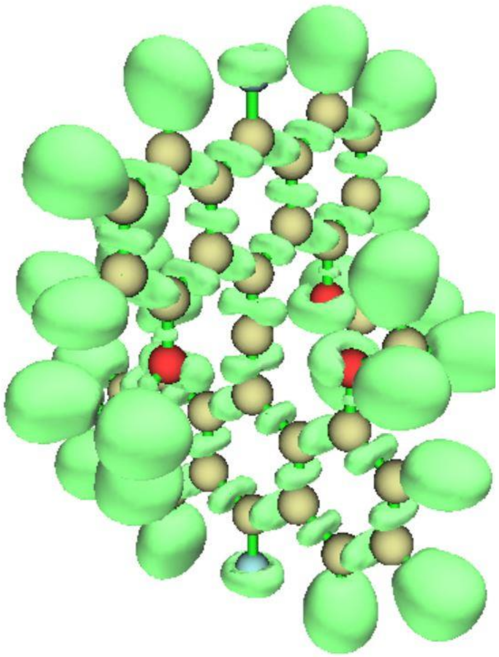

Figure 3: Electron localization function for the D2 variant with the >CF(+) bridge

NCI analysis

The plot of s (also called the RDG, reduced density gradient) shows a spike characteristic of NCI at values of λ_2-signed density near -0.02 and several spikes in the +0.015 to +0.025 interval.

The NCI zones include the centers of aromatic rings, interactions between each F and the hydrogens on adjacent anthryl Carbons, and the methoxy methyl H atoms and the hydrogens on adjacent anthryl Carbons. The zones of central importance lie between the hcC and the four “equatorial” methoxy Oxygen atoms in its coordination sphere. This reinforces further the inference that the central C is not hexavalent.

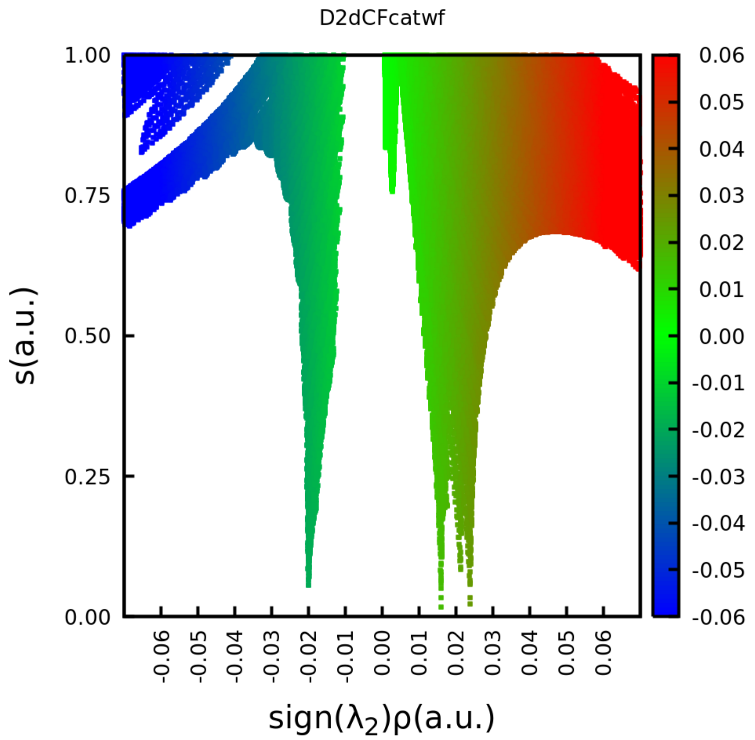

Figure 4: Identifier of zones with small signed density and small RDG for the CF(+) bridged D2d variant

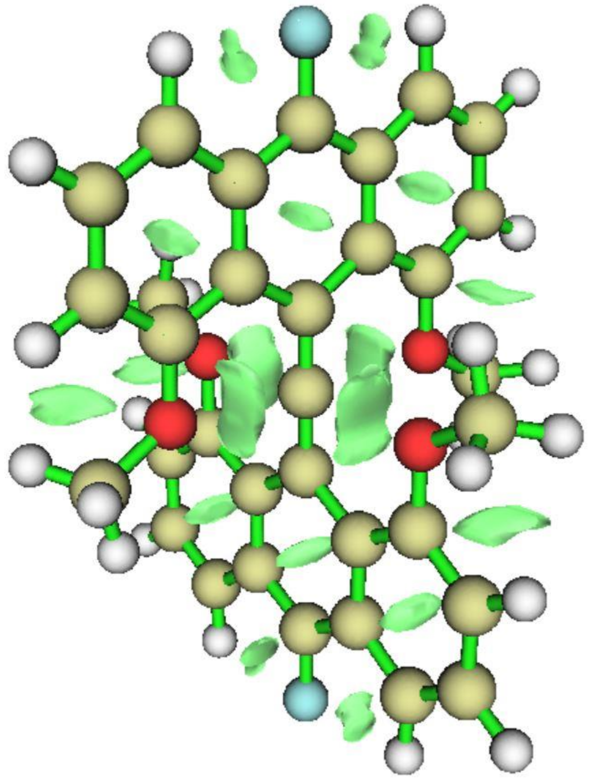

Figure 5: NCI for CF(+) variant

D_{2h} spiropentadiene dication: Plot of RDG vs λ2-signed density

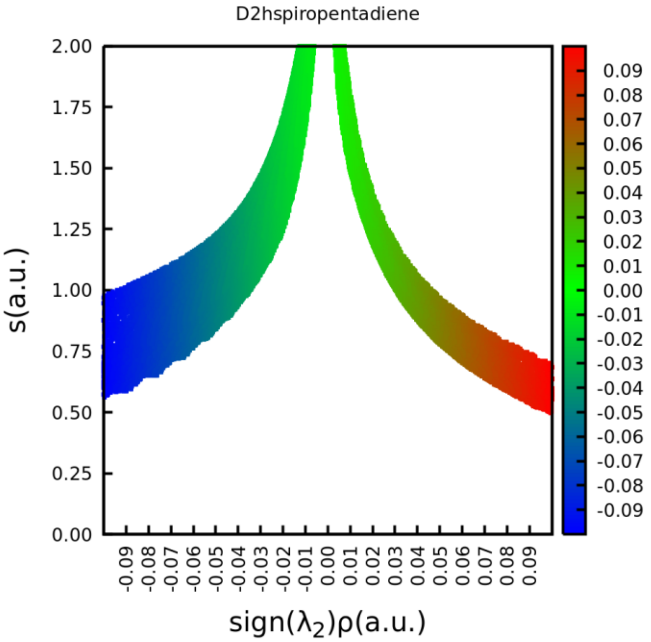

Figure 6: RDG vs sign(λ2)ρ for spiropentadiene dication

The structure of the RDG plot against Sign(λ2)ρ shows that there are no regions of non-covalent interaction.

Hexamethyl Benzene dication: Plot of RDG vs λ2-signed density

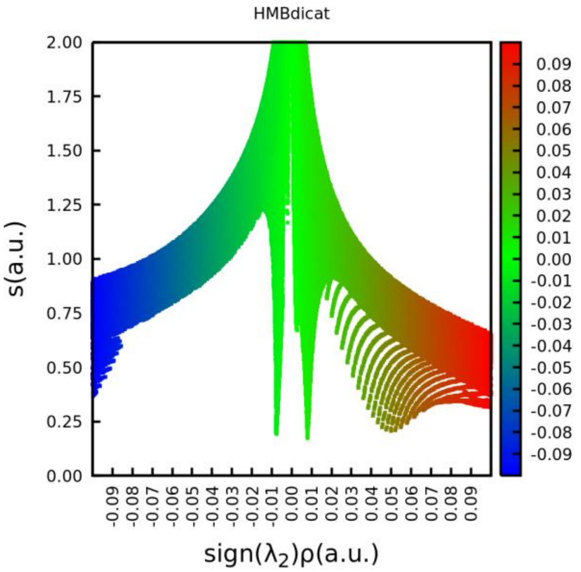

Figure 7:RDG vs λ2-signed density shows two non-covalent interactions

Radom's dimethanospiro[2.2]octaplane dication

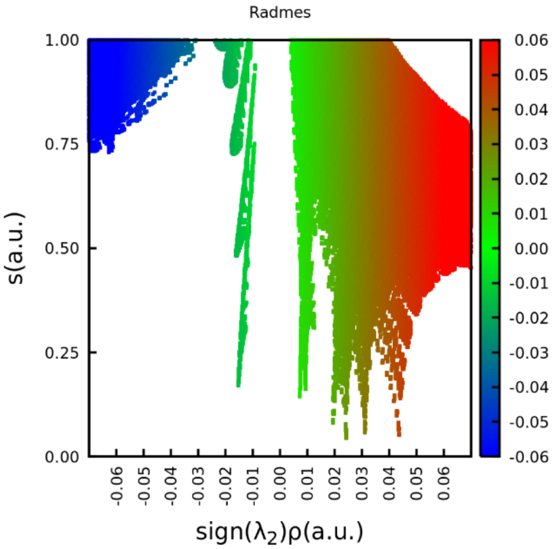

Figure 8: RDG vs λ2-signed density for Radom's dimethanospiro[2.2]octaplane dication shows a number of weak interactions

Yamaguchi's five-coordinate carbon compound: Plot of RDG vs λ2-signed density

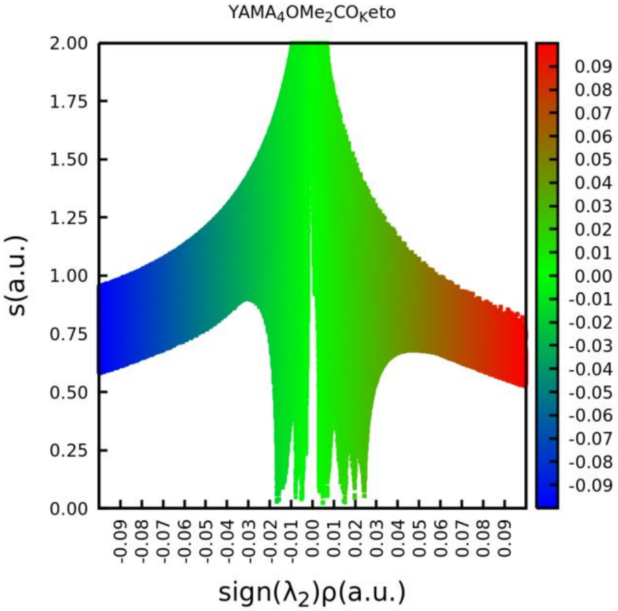

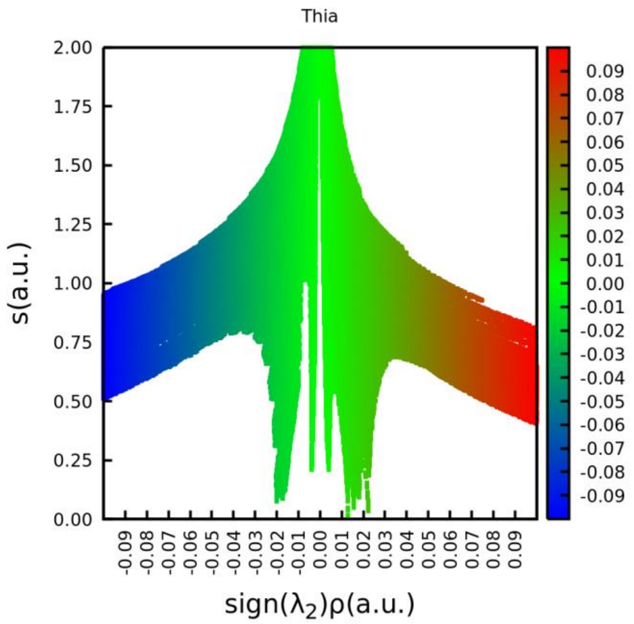

Figure 9: RDG vs λ2-signed density for 1,8-dimethoxy-9-dimethoxymethylanthracene cation (top) and its thia-variant (bottom) show a number of weak interactions

Further depiction of the (–SMe, >C=O) system

A contour diagram of ELF in the S….hcC…C(allene) plane (Figure 1S) shows a depletion of charge between the thiomethyl S and the hcC, indicating a non-covalent interaction.

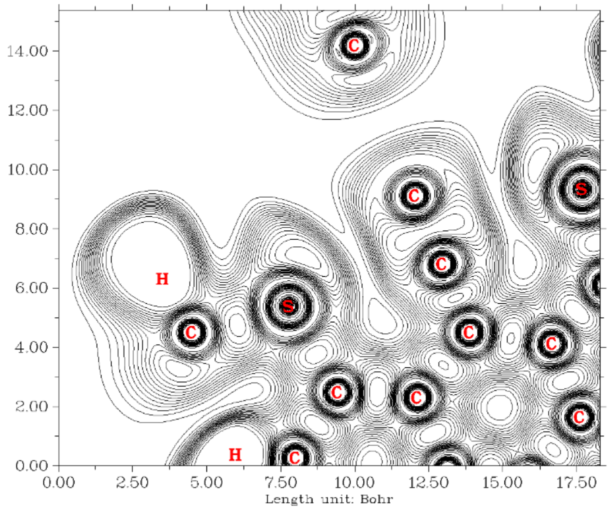

Figure 10: NCI contour plot for D_{2d} species

ELF of Conventional bonds – the allene fragment:
The figure below shows the ELF along the line connecting the three C atoms of the central allene fragment. ELF reaches its maximum (unity) at nuclear centers. Red arrows identify the three C atoms in the core allene. For very well established bonds such as the C=C bonds, the ELF passes through a maximum at the bond CP. These are shown by blue arrows

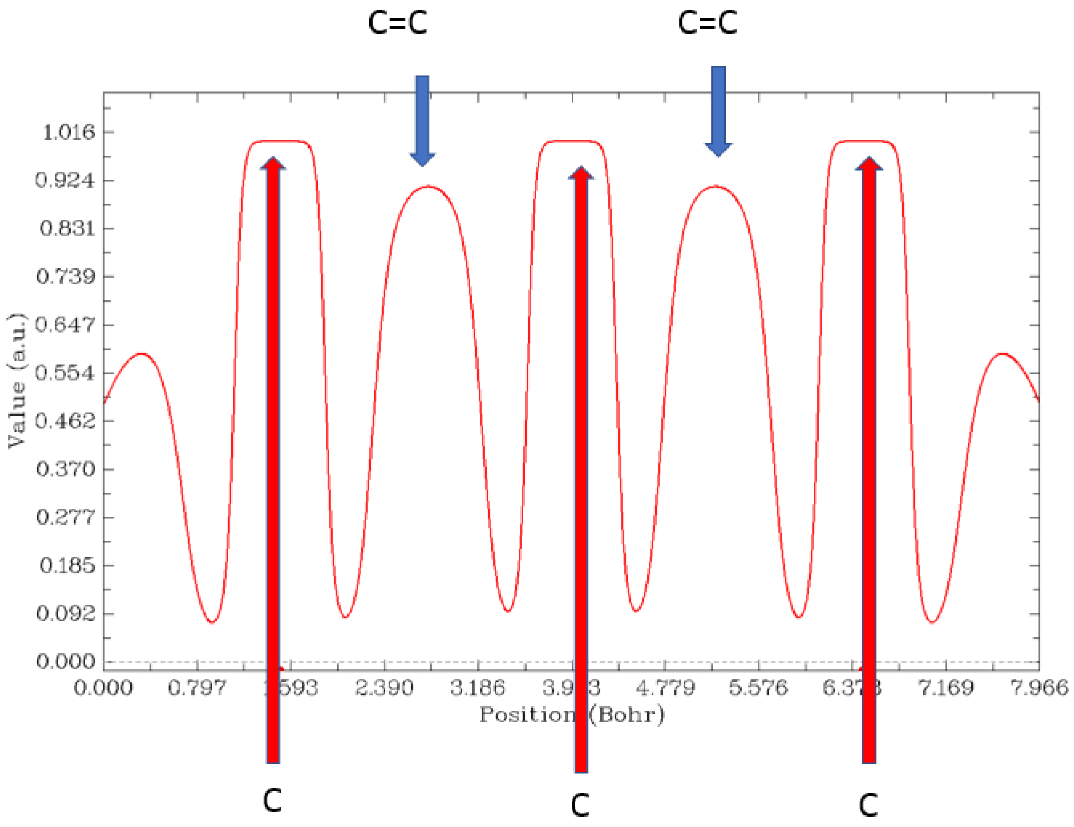

Figure 11: ELF function along the line connecting the allene carbons

ELF for unconventional interactions:
We get a very different view from the ELF along the line between coordinated S atoms, which passes through the allene zone. ELF shows the shell structure for second-row S, and the pi distribution near the central Carbon. Between S and the allene zone (showing some pi character) we see the localization associated with the S atom lone pair, and depletion of charge closer to allene.

The ELF along the line from S to the central C (Figure 8) shows the shell structure of S (left, blue arrow) and the simpler C atom attractor (right, black arrow). The electrostatic interaction is indicated by depletion of charge between the atoms (red arrow).

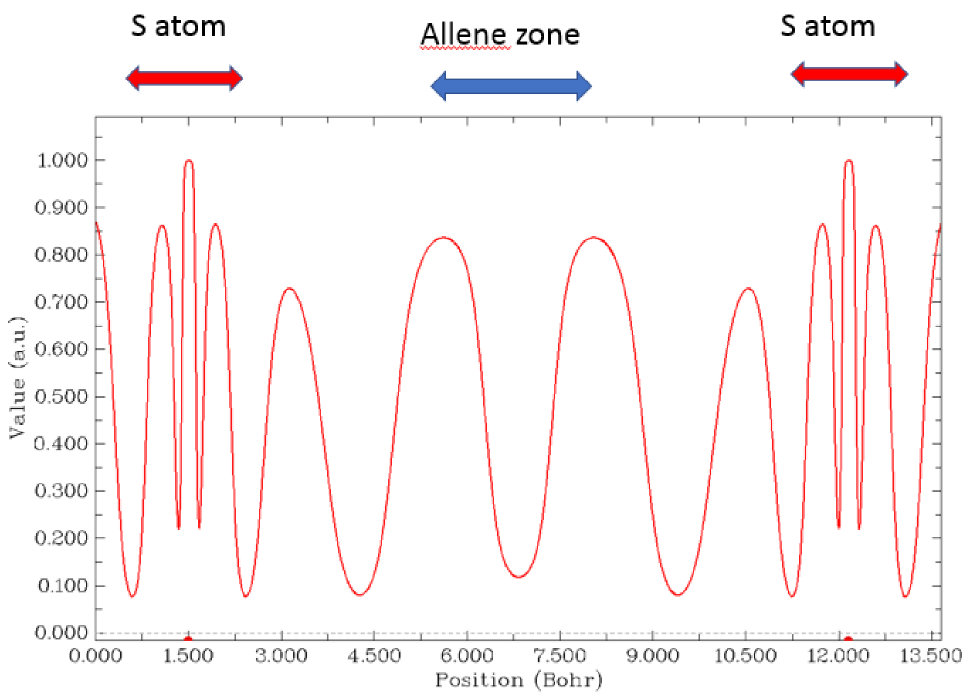

Figure 12: ELF function along the line S through allene to S

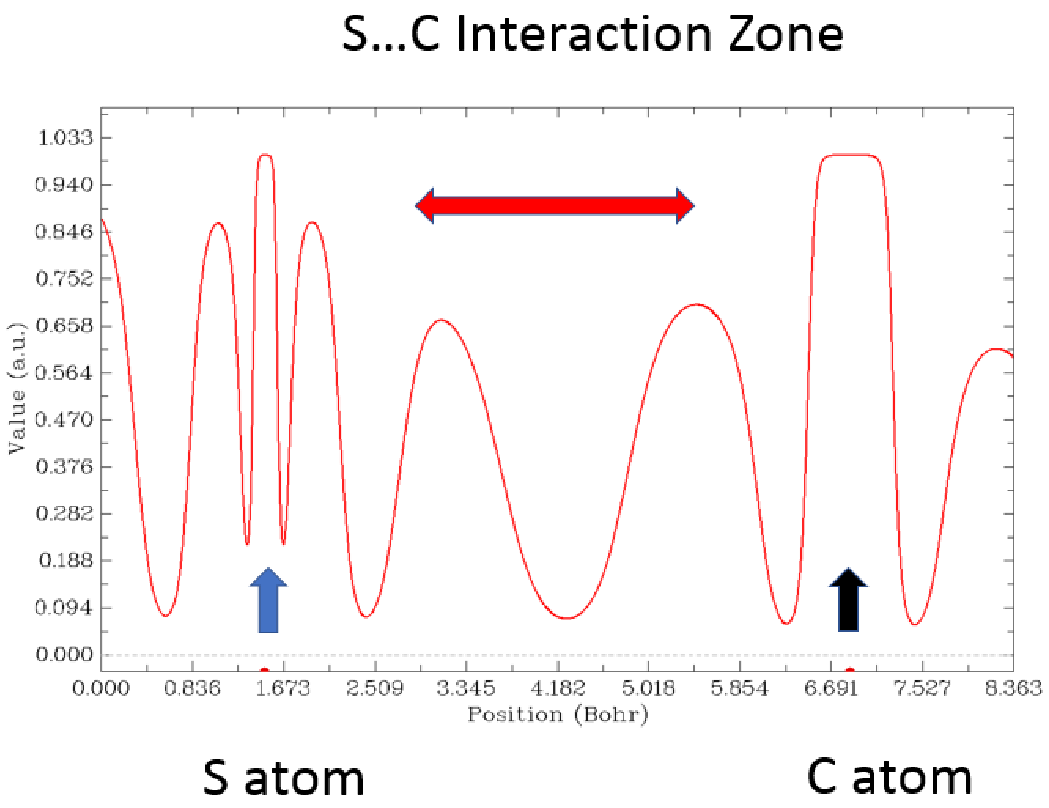

Figure 13: ELF on the line from S to the allene central carbon

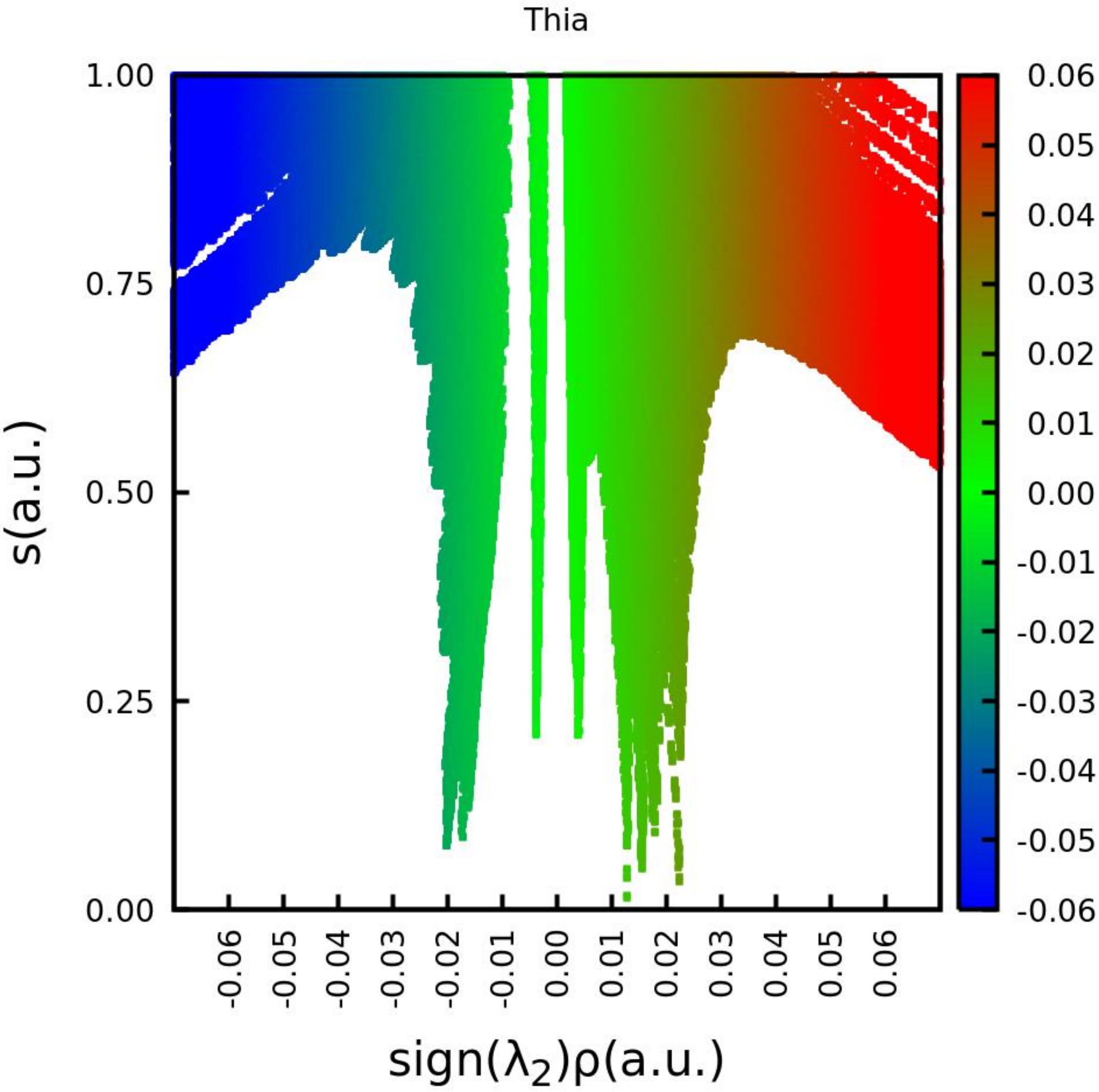

Figure 14: Plot RDG vs λ2-signed density for the thia variant of show a number of weak interactions

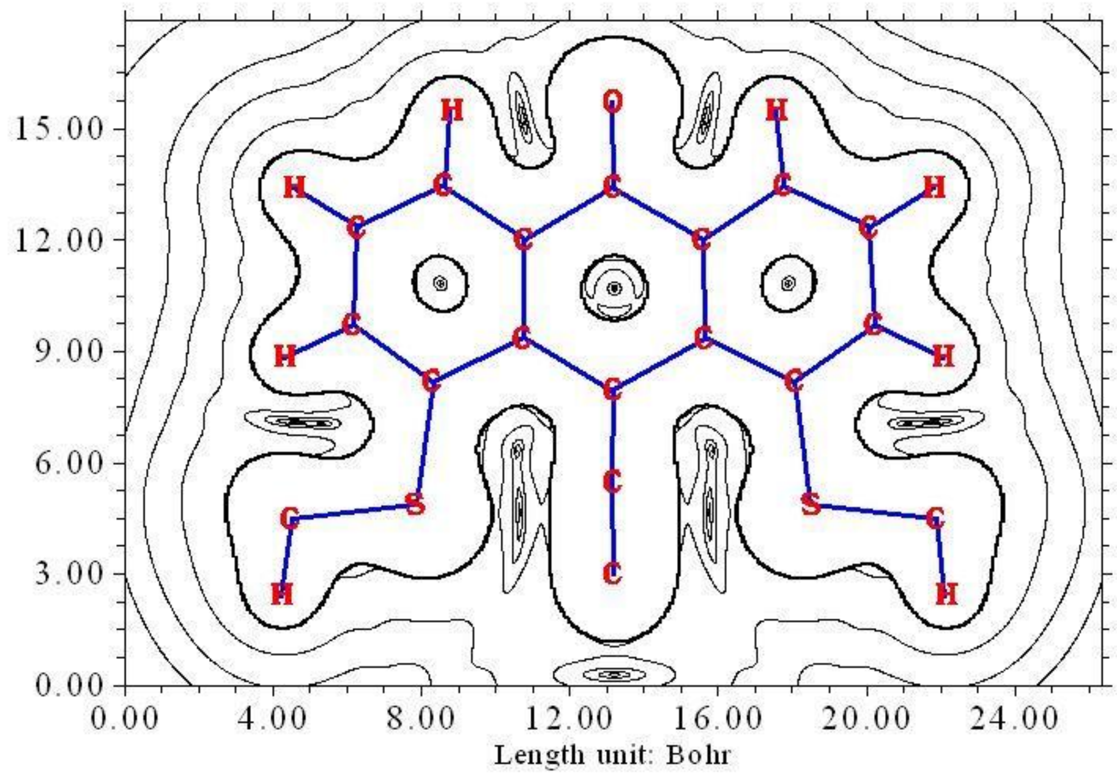

Figure 15: Contour diagram of NCI in one anthryl plane

The contour plot (Figure 15) gives an alternative view of these NCI regions. Note the prominent feature between the allyl carbons and the CH_3 – S – substituents on the anthryl moiety in the plane of the contours.

Once again small non-covalent interaction (NCI) zones are to be found between carbonyl oxygens and hydrogen atoms of nearby phenyl ring CH fragments. Bader analysis identifies no CPs there. NCI zones are shown in the centers of the three fused six-membered rings of the anthryl fragment, where Bader analysis identifies ring CPs. NCI zones are located between –SMe methyl CH bonds and ring CH bonds. Major NCI zones are located between the four –SMe Sulfur atoms and the central allenyl Carbon atom.

Neutral symmetric systems: >SO_2 bridge (S2)

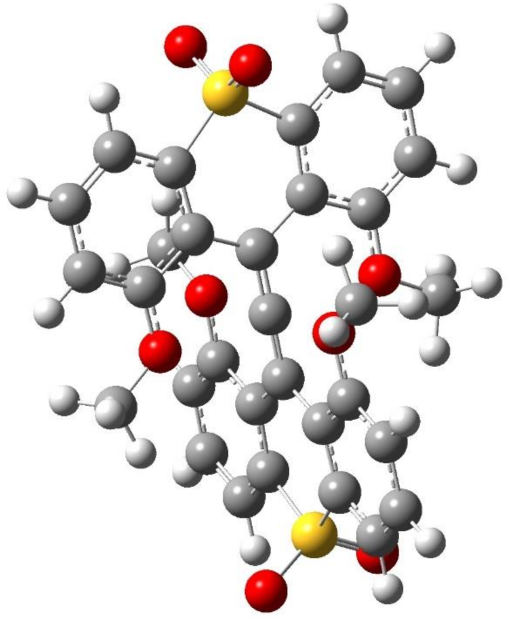

Figure 16: Structure of the >SO_2 bridged variant of Yamaguchi's hcC system

AIM analysis

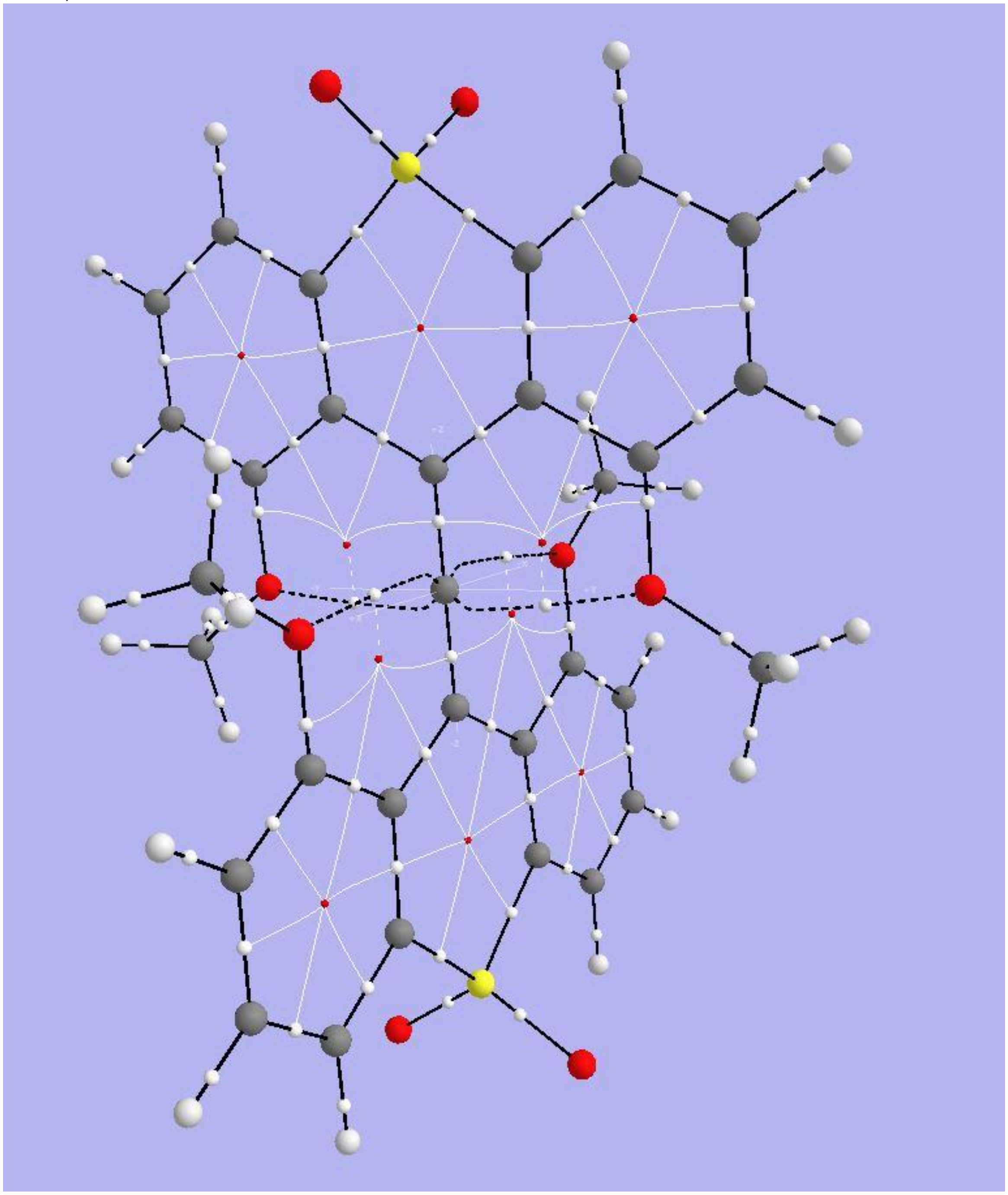

Figure 17: AIM critical points and bond paths for >SO2 bridged variant of Yamaguchi's hcC system

Critical bond points shown in Figure 17 for the allyl double bonds, the S-O bond and the Methyl -O bonds are indicative of covalent bonding. The BCP parameters (Table 2) for the interaction between the hcC and the methoxy oxygen are characteristic of non-covalent bonding; ionic in this case.

Critical Point	ρ	Laplacian	G	V	|2G/V|
C=C bond (3,-1)	0.3608	-1.2384	+0.1881	-0.6859	0.3158
O-S bond (3,-1)	0.3051	-0.4285	+0.3631	-0.8333	0.8715
H_3C-O bond (3,-1)	0.2575	-0.3539	+0.2796	-0.6476	0.8635
MeO … C (3,-1)	0.0221	+0.0884	0.0196	-0.0171	2.2924

Table 2: Bader parameters for the >SO2 - bridged neutral variant of Yamaguchi's hcC compound

Charges (absolute electrons) on selected atomic basis are listed below

hcC	Allene C	Coordinated O	Bridging S	O on bridging C
-0.3032	+0.2188	-1.1744	+2.7211	-1.3863

ELF analysis

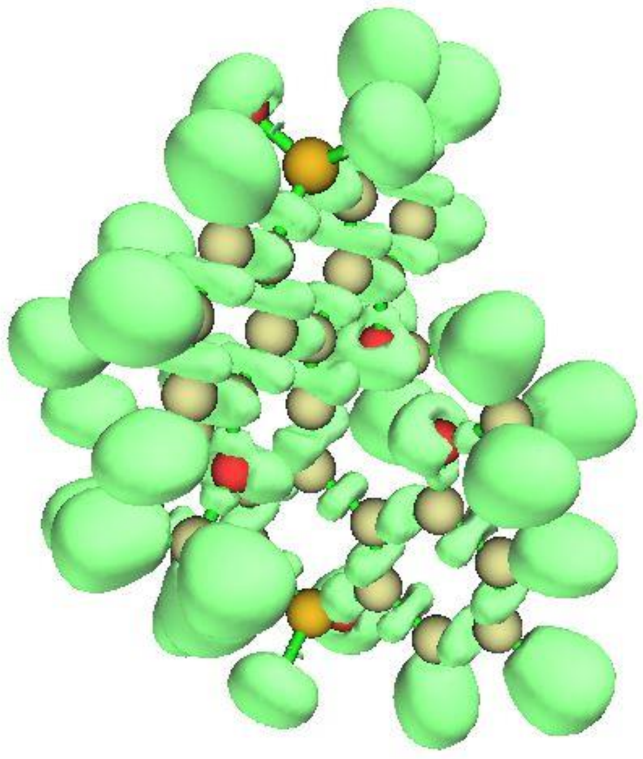

Figure 18: ELF isosurface for the >SO2 bridged variant of Yamaguchi's hcC compound

Figure 18 displays the ELF isosurfaces for the >SO_2 bridged variant of Yamaguchi's six-coordinate system. We see zones of localized charge near the SO_2 oxygens and enclosing the CH

bonds. ELF zones between methoxy oxygens and the hcC carbon are absent, indicating that there is no covalent connection between the hcC and the methoxy oxygens in its coordination sphere.

NCI analysis

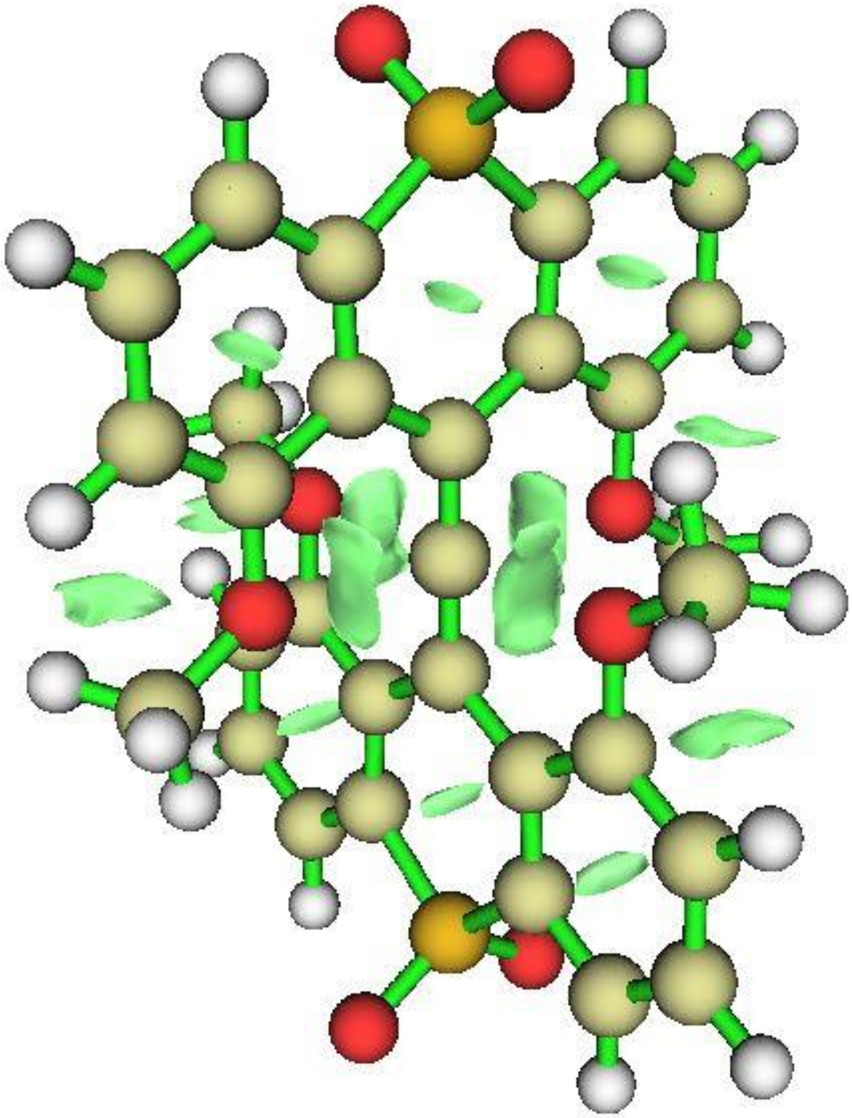

Figure 19: NCI zones for the >SO_2 – bridged variant of Yamaguchi's hcC system

As we see in all the symmetric variants of Yamaguchi's hcC system, there are non-covalent interactions in the center of six-membered rings and between the Methoxy groups and the adjacent CH of the anthryl rings. The most prominent features however are the four NCI zones between methoxy oxygens and the hcC.

Response to enhanced basis set

Tables 3-8 contain Bader parameters for **6** and **7**, with cc-pVTZ and aug-cc-pVTZ basis sets. These quantities are almost unchanged for **6**, but show noticeable shifts for charges for S-bearing **7**. The qualitative picture – that the electrostatic interaction between OMe and hcC is repulsive while the electrostatic interaction between SMe and hcC is attractive – is unchanged.

Table 3: Selected Bader parameters for D2d variant (6) with >C=O bridging (cc-pVTZ)

Critical Point	ρ	Laplacian	G	V	∣2G/V∣
C=O bond (3,-1)	0.4139	-0.2747	+0.6524	-1.3736	0.9499
C=C bond (3,-1)	0.3600	-1.2359	+0.1899	-0.6888	0.5514
H_3C-O bond (3,-1)	0.2602	-0.3771	+0.2787	-0.6517	0.8553
MeO … C (3,-1)	0.0201	+0.0802	0.0176	-0.0151	2.3311

Table 4: Selected Bader parameters for D2d variant (6) with >C=O bridging (aug-cc-pVTZ)

Critical Point	ρ	Laplacian	G	V	∣2G/V∣
C=O bond (3,-1)	0.4144	-0.2747	+0.6566	-1.3819	0.9503
C=C bond (3,-1)	0.3600	-1.2324	+0.1905	-0.6890	0.5530
H_3C-O bond (3,-1)	0.2600	-0.3500	+0.2848	-0.6571	0.8668
MeO … C (3,-1)	0.0203	+0.0793	0.0176	-0.0153	2.3006

Table 5: AIM Charges for atoms in the hcC coordination zone for species 6 and 7. (cc-pVTZ)

Charges (O)	C(allyl)	hcC	O atom
Species **6**	+0.2193	-0.3279	-1.1729
Charges (S)	C(allyl)	hcC	S atom
Species **7**	+0.1552	-0.3484	+0.1130

Table 6: AIM Charges for atoms in the hcC coordination zone for species 6 and 7. (aug-cc-pVTZ)

Charges (O)	C(allyl)	hcC	O atom
Species **6**	+0.2213	-0.3256	-1.1815
Charges (S)	C(allyl)	hcC	S atom
Species **7**	+0.2077	-0.4591	+0.1087

Table 7: AIM Charges for atoms in the hcC coordination zone for species 6 and 7. (aug-cc-pVTZ)

Critical Point	ρ	Laplacian	G	V	∣2G/V∣

C=O bond (3,-1)	0.4095	-0.0281	+0.6700	-1.3470	0.9948
C=C bond (3,-1)	0.3448	-1.0315	+0.1624	-0.5826	0.5575
H_3C-S bond (3,-1)	0.1845	-0.2965	+0.0497	-0.1736	0.5726
MeS … hcC (3,-1)	0.0201	+0.0619	+0.0141	-0.0128	2.3125

Table 8: Selected Bader parameters for D2d thia methyl variant (7) with >C=O bridging (aug-cc-pVTZ)

Critical Point	ρ	Laplacian	G	V	$\lvert 2G/V \rvert$
C=O bond (3,-1)	0.4199	-0.2625	+0.6610	-1.3876	0.9527
C=C bond (3,-1)	0.3594	-1.2388	+0.1826	-0.6753	0.5408
H_3C-S bond (3,-1)	0.1856	-0.3392	+0.0511	-0.1765	0.5790
MeS … hcC (3,-1)	0.0206	+0.0596	+0.0142	-0.0135	2.1037

Coordinates for systems studied

Spiropentadiene dication (1)

1 C1 0.0000 0.6408 1.3338 C
2 H2 0.0000 1.6732 1.6834 H
3 C3 0.0000 0.6408 -1.3338 C
4 H4 0.0000 1.6732 -1.6834 H
5 C5 0.0000 -0.6408 -1.3338 C
6 H6 0.0000 -1.6732 -1.6834 H
7 C7 0.0000 -0.6408 1.3338 C
8 H8 0.0000 -1.6732 1.6834 H
9 C9 0.0000 0.0000 0.0000 C

Hexamethyl benzene dication (2)

1 C1 1.2186 0.1568 -0.4725 C
2 C2 0.2280 1.2075 -0.4713 C
3 C3 -1.0785 0.5891 -0.4671 C
4 C4 -0.8951 -0.8437 -0.4727 C
5 C5 0.5244 -1.1110 -0.4677 C
6 C6 0.0001 0.0004 0.7043 C
7 C7 0.0026 0.0006 2.1952 C

8 H8 0.9987 -0.2481 2.5650 H
9 H9 -0.2800 0.9867 2.5674 H
10 H10 -0.7094 -0.7376 2.5679 H
11 C11 0.4922 2.6607 -0.2575 C
12 H12 0.1878 3.1906 -1.1641 H
13 H13 -0.0908 3.0602 0.5735 H
14 H14 1.5475 2.8609 -0.0830 H
15 C15 -2.3800 1.2892 -0.2522 C
16 H16 -2.2433 2.3564 -0.0879 H
17 H17 -2.9833 1.1572 -1.1541 H
18 H18 -2.9334 0.8659 0.5870 H
19 C19 2.6823 0.3544 -0.2560 C
20 H20 3.0914 0.8277 -1.1525 H
21 H21 2.8806 1.0140 0.5901 H
22 H22 3.2007 -0.5900 -0.1025 H
23 C23 1.1674 -2.4416 -0.2541 C
24 H24 1.7220 -2.6932 -1.1619 H
25 H25 1.8748 -2.4208 0.5759 H
26 H26 0.4320 -3.2239 -0.0766 H
27 C27 -1.9631 -1.8629 -0.2571 C
28 H28 -1.7214 -2.5340 0.5684 H
29 H29 -2.9324 -1.4036 -0.0737 H
30 H30 -2.0351 -2.4655 -1.1666 H

Dimethanospiro[2.2]octaplane dication (3)

1 C1 1.3004 1.3794 1.3094 C
2 C2 0.0000 2.2595 1.5115 C
3 C3 -1.3004 1.3794 1.3094 C
4 C4 -0.7144 0.0000 1.3348 C
5 C5 0.7144 0.0000 1.3348 C
6 C6 -1.3004 -1.3794 1.3094 C
7 C7 0.0000 -2.2595 1.5115 C
8 C8 1.3004 -1.3794 1.3094 C
9 H9 1.9825 1.5470 2.1427 H
10 H10 0.0000 2.5579 2.5582 H
11 H11 -1.9825 -1.5470 2.1427 H
12 H12 0.0000 -3.1806 0.9426 H
13 H13 1.9825 -1.5470 2.1427 H
14 H14 2.8654 2.1841 0.0000 H
15 H15 3.6228 0.0000 -0.8717 H
16 H16 3.6228 0.0000 0.8717 H
17 H17 2.8654 -2.1841 0.0000 H
18 H18 -2.8654 -2.1841 0.0000 H

19 H19	-2.8654	2.1841	0.0000	H
20 H20	-3.6228	0.0000	0.8717	H
21 H21	-3.6228	0.0000	-0.8717	H
22 H22	0.0000	3.1806	0.9426	H
23 C23	1.3004	1.3794	-1.3094	C
24 C24	0.0000	2.2595	-1.5115	C
25 C25	-1.3004	1.3794	-1.3094	C
26 C26	-0.7144	0.0000	-1.3348	C
27 C27	0.7144	0.0000	-1.3348	C
28 H28	1.9825	1.5470	-2.1427	H
29 H29	0.0000	3.1806	-0.9426	H
30 H30	-1.9825	1.5470	-2.1427	H
31 C31	-1.3004	-1.3794	-1.3094	C
32 H32	-1.9825	-1.5470	-2.1427	H
33 C33	1.3004	-1.3794	-1.3094	C
34 H34	1.9825	-1.5470	-2.1427	H
35 C35	0.0000	-2.2595	-1.5115	C
36 H36	0.0000	-2.5579	-2.5582	H
37 H37	0.0000	-3.1806	-0.9426	H
38 H38	0.0000	-2.5579	2.5582	H
39 C39	0.0000	0.0000	0.0000	C
40 C40	2.9663	0.0000	0.0000	C
41 C41	2.1499	1.3642	0.0000	C
42 C42	2.1499	-1.3642	0.0000	C
43 C43	-2.1499	1.3642	0.0000	C
44 C44	-2.1499	-1.3642	0.0000	C
45 C45	-2.9663	0.0000	0.0000	C
46 H46	-1.9825	1.5470	2.1427	H
47 H47	0.0000	2.5579	-2.5582	H

1,8-dimethoxy-9-dimethoxy anthracene cation (**4**)

1 C1	0.0000	3.6686	0.7135	C
2 C2	0.0000	2.5079	-0.0021	C
3 C3	0.0000	1.2276	0.6521	C
4 C4	0.0000	1.2191	2.0818	C
5 C5	0.0000	2.4533	2.8007	C
6 C6	0.0000	3.6350	2.1344	C
7 C7	0.0000	0.0000	-0.0424	C
8 C8	0.0000	0.0000	2.7510	C
9 C9	0.0000	-1.2191	2.0818	C
10 C10	0.0000	-1.2276	0.6521	C
11 C11	0.0000	-2.5079	-0.0021	C
12 C12	0.0000	-3.6686	0.7135	C

13 C13	0.0000	-3.6350	2.1344	C
14 C14	0.0000	-2.4533	2.8007	C
15 H15	0.0000	4.6279	0.2118	H
16 H16	0.0000	2.4239	3.8845	H
17 H17	0.0000	4.5730	2.6774	H
18 H18	0.0000	0.0000	3.8370	H
19 H19	0.0000	-4.6279	0.2118	H
20 H20	0.0000	-4.5730	2.6774	H
21 H21	0.0000	-2.4239	3.8845	H
22 O22	0.0000	2.4286	-1.3534	O
23 O23	0.0000	-2.4286	-1.3534	O
24 C24	0.0000	3.6200	-2.1254	C
25 H25	0.8960	4.2123	-1.9218	H
26 H26	0.0000	3.3015	-3.1652	H
27 H27	-0.8960	4.2123	-1.9218	H
28 C28	0.0000	-3.6200	-2.1254	C
29 H29	-0.8960	-4.2123	-1.9218	H
30 H30	0.0000	-3.3015	-3.1652	H
31 H31	0.8960	-4.2123	-1.9218	H
32 C32	0.0000	0.0000	-1.5361	C
33 O33	1.0637	0.0000	-2.2410	O
34 O34	-1.0637	0.0000	-2.2410	O
35 C35	2.3573	0.0000	-1.5965	C
36 H36	2.4628	-0.8990	-0.9917	H
37 H37	3.0715	0.0000	-2.4136	H
38 H38	2.4628	0.8990	-0.9917	H
39 C39	-2.3573	0.0000	-1.5965	C
40 H40	-2.4628	-0.8990	-0.9917	H
41 H41	-2.4628	0.8990	-0.9917	H
42 H42	-3.0715	0.0000	-2.4136	H

Neutral CO bridged -OCH_3 variant of Yamaguchi's hexacoordinated carbon compound (6)

1 C1	0.0000	-3.7070	2.1827	C
2 C2	0.0000	-2.5410	1.4350	C
3 C3	0.0000	-1.2732	2.0680	C
4 C4	0.0000	-1.2679	3.4655	C
5 C5	0.0000	-2.4476	4.2101	C
6 C6	0.0000	-3.6612	3.5710	C
7 C7	0.0000	0.0000	1.3142	C
8 C8	0.0000	1.2679	3.4655	C
9 C9	0.0000	1.2732	2.0680	C
10 C10	0.0000	2.5410	1.4350	C
11 C11	0.0000	3.7070	2.1827	C

12	C12	0.0000	3.6612	3.5710	C
13	C13	0.0000	2.4476	4.2101	C
14	H14	0.0000	-4.6645	1.6849	H
15	H15	0.0000	-2.3679	5.2867	H
16	H16	0.0000	-4.5827	4.1365	H
17	H17	0.0000	4.6645	1.6849	H
18	H18	0.0000	4.5827	4.1365	H
19	H19	0.0000	2.3679	5.2867	H
20	C20	0.0000	0.0000	0.0000	C
21	C21	0.0000	0.0000	-1.3142	C
22	C22	-1.2732	0.0000	-2.0680	C
23	C23	-1.2679	0.0000	-3.4655	C
24	C24	-2.5410	0.0000	-1.4350	C
25	C25	-3.7070	0.0000	-2.1827	C
26	C26	-3.6612	0.0000	-3.5710	C
27	H27	-4.6645	0.0000	-1.6849	H
28	H28	-4.5827	0.0000	-4.1365	H
29	C29	1.2732	0.0000	-2.0680	C
30	C30	1.2679	0.0000	-3.4655	C
31	C31	2.5410	0.0000	-1.4350	C
32	C32	2.4476	0.0000	-4.2101	C
33	C33	3.7070	0.0000	-2.1827	C
34	C34	3.6612	0.0000	-3.5710	C
35	H35	2.3679	0.0000	-5.2867	H
36	H36	4.6645	0.0000	-1.6849	H
37	H37	4.5827	0.0000	-4.1365	H
38	H38	-2.3679	0.0000	-5.2867	H
39	C39	-3.7721	0.0000	0.6067	C
40	H40	-4.3623	0.8922	0.3823	H
41	H41	-4.3623	-0.8922	0.3823	H
42	H42	-3.5096	0.0000	1.6614	H
43	C43	0.0000	3.7721	-0.6067	C
44	H44	-0.8922	4.3623	-0.3823	H
45	H45	0.8922	4.3623	-0.3823	H
46	H46	0.0000	3.5096	-1.6614	H
47	C47	3.7721	0.0000	0.6067	C
48	H48	4.3623	-0.8922	0.3823	H
49	H49	4.3623	0.8922	0.3823	H
50	H50	3.5096	0.0000	1.6614	H
51	C51	0.0000	-3.7721	-0.6067	C
52	H52	0.8922	-4.3623	-0.3823	H
53	H53	-0.8922	-4.3623	-0.3823	H
54	H54	0.0000	-3.5096	-1.6614	H
55	C55	-2.4476	0.0000	-4.2101	C

56 C56	0.0000	0.0000	4.2296	C
57 C57	0.0000	0.0000	-4.2296	C
58 O58	0.0000	0.0000	-5.4453	O
59 O59	0.0000	0.0000	5.4453	O
60 O60	-2.5514	0.0000	-0.0902	O
61 O61	0.0000	2.5514	0.0902	O
62 O62	0.0000	-2.5514	0.0902	O
63 O63	2.5514	0.0000	-0.0902	O

Neutral CO bridged -SCH_3 variant of Yamaguchi's hexacoordinated carbon compound (7)

1 C1	-2.6290	2.6287	2.2451	C
2 C2	-1.8275	1.8275	1.4382	C
3 C3	-0.9169	0.9168	2.0508	C
4 C4	-0.8980	0.8976	3.4521	C
5 C5	-1.7122	1.7113	4.2311	C
6 C6	-2.5814	2.5805	3.6258	C
7 C7	0.0000	0.0000	1.3129	C
8 C8	0.8980	-0.8976	3.4521	C
9 C9	0.9169	-0.9168	2.0508	C
10 C10	1.8275	-1.8275	1.4382	C
11 C11	2.6290	-2.6287	2.2451	C
12 C12	2.5814	-2.5805	3.6258	C
13 C13	1.7122	-1.7113	4.2311	C
14 H14	-3.3203	3.3201	1.7943	H
15 H15	-1.6303	1.6292	5.3041	H
16 H16	-3.2247	3.2235	4.2102	H
17 H17	3.3203	-3.3201	1.7943	H
18 H18	3.2247	-3.2235	4.2102	H
19 H19	1.6303	-1.6292	5.3041	H
20 C20	0.0000	0.0000	0.0000	C
21 C21	0.0000	0.0000	-1.3129	C
22 C22	0.9169	0.9168	-2.0508	C
23 C23	0.8980	0.8976	-3.4521	C
24 C24	1.8275	1.8275	-1.4382	C
25 C25	2.6290	2.6287	-2.2451	C
26 C26	2.5814	2.5805	-3.6258	C
27 H27	3.3203	3.3201	-1.7943	H
28 H28	3.2247	3.2235	-4.2102	H
29 C29	-0.9169	-0.9168	-2.0508	C
30 C30	-0.8980	-0.8976	-3.4521	C
31 C31	-1.8275	-1.8275	-1.4382	C
32 C32	-1.7122	-1.7113	-4.2311	C
33 C33	-2.6290	-2.6287	-2.2451	C

34 C34	-2.5814	-2.5805	-3.6258	C
35 H35	-1.6303	-1.6292	-5.3041	H
36 H36	-3.3203	-3.3201	-1.7943	H
37 H37	-3.2247	-3.2235	-4.2102	H
38 H38	1.6303	1.6292	-5.3041	H
39 C39	3.2657	3.2662	0.5205	C
40 H40	4.2248	2.9592	0.1080	H
41 H41	2.9593	4.2251	0.1071	H
42 H42	3.3661	3.3674	1.5994	H
43 C43	3.2657	-3.2662	-0.5205	C
44 H44	4.2248	-2.9592	-0.1080	H
45 H45	2.9593	-4.2251	-0.1071	H
46 H46	3.3661	-3.3674	-1.5994	H
47 C47	-3.2657	-3.2662	0.5205	C
48 H48	-4.2248	-2.9592	0.1080	H
49 H49	-2.9593	-4.2251	0.1071	H
50 H50	-3.3661	-3.3674	1.5994	H
51 C51	-3.2657	3.2662	-0.5205	C
52 H52	-4.2248	2.9592	-0.1080	H
53 H53	-2.9593	4.2251	-0.1071	H
54 H54	-3.3661	3.3674	-1.5994	H
55 C55	1.7122	1.7113	-4.2311	C
56 C56	0.0000	0.0000	4.2086	C
57 C57	0.0000	0.0000	-4.2086	C
58 O58	0.0000	0.0000	-5.4235	O
59 O59	0.0000	0.0000	5.4235	O
60 S60	1.9982	-1.9989	-0.3211	S
61 S61	-1.9982	-1.9989	0.3211	S
62 S62	-1.9982	1.9989	-0.3211	S
63 S63	1.9982	1.9989	0.3211	S

Dicationic CF bridged -OCH_3 variant of Yamaguchi's hexacoordinated carbon compound (**S1**)

1 C1	2.1581	3.0823	1.9536	C
2 C2	1.4743	2.0809	1.3406	C
3 C3	2.1355	1.0465	0.6392	C
4 C4	3.5509	1.0403	0.6127	C
5 C5	4.2534	2.1086	1.2500	C
6 C6	3.5733	3.0850	1.8882	C
7 C7	1.3812	0.0007	-0.0012	C
8 C8	3.5520	-1.0377	-0.6135	C
9 C9	2.1368	-1.0448	-0.6405	C
10 C10	1.4763	-2.0800	-1.3414	C
11 C11	2.1607	-3.0812	-1.9539	C

12 C12	3.5759	-3.0829	-1.8883	C
13 C13	4.2553	-2.1057	-1.2503	C
14 H14	1.6507	3.8567	2.4873	H
15 H15	5.3216	2.1029	1.2103	H
16 H16	4.1071	3.8819	2.3681	H
17 H17	1.6538	-3.8562	-2.4873	H
18 H18	4.1104	-3.8795	-2.3679	H
19 H19	5.3235	-2.0994	-1.2105	H
20 C20	0.0339	0.0004	-0.0029	C
21 C21	-1.3659	-0.0003	-0.0005	C
22 C22	-2.0840	1.0736	-0.5975	C
23 C23	-3.5035	1.0802	-0.5468	C
24 C24	-1.4576	2.1859	-1.2789	C
25 C25	-2.2101	3.1934	-1.7825	C
26 C26	-3.6245	3.1902	-1.6734	C
27 H27	-1.7407	4.0098	-2.2872	H
28 H28	-4.1758	4.0139	-2.0807	H
29 C29	-2.0812	-1.0754	0.5976	C
30 C30	-3.5008	-1.0849	0.5485	C
31 C31	-1.4519	-2.1861	1.2791	C
32 C32	-4.2563	-2.1724	1.0855	C
33 C33	-2.2018	-3.1950	1.7835	C
34 C34	-3.6163	-3.1950	1.6754	C
35 H35	-5.3211	-2.1404	1.0043	H
36 H36	-1.7302	-4.0102	2.2882	H
37 H37	-4.1655	-4.0198	2.0833	H
38 H38	-5.3265	2.1319	-1.0009	H
39 O39	-0.1092	2.1414	-1.3978	O
40 O40	-0.1035	-2.1384	1.3974	O
41 O41	0.1186	1.9393	1.3516	O
42 O42	0.1206	-1.9389	-1.3521	O
43 C43	0.6353	3.1565	-2.0945	C
44 H44	0.3320	3.2165	-3.1319	H
45 H45	0.5114	4.1222	-1.6212	H
46 H46	1.6625	2.8441	-2.0236	H
47 C47	-0.7236	-2.7759	-2.1630	C
48 H48	-0.4290	-2.7197	-3.2022	H
49 H49	-0.6819	-3.8024	-1.8243	H
50 H50	-1.7199	-2.3890	-2.0353	H
51 C51	0.6437	-3.1514	2.0942	C
52 H52	0.3409	-3.2118	3.1317	H
53 H53	0.5218	-4.1175	1.6212	H
54 H54	1.6701	-2.8366	2.0227	H
55 C55	-0.7257	2.7765	2.1622	C

56 H56 -0.4313 2.7205 3.2014 H
57 H57 -0.6839 3.8029 1.8234 H
58 H58 -1.7220 2.3895 2.0344 H
59 C59 -4.2618 2.1662 -1.0830 C
60 C60 4.2157 0.0016 -0.0004 C
61 C61 -4.1487 -0.0031 0.0011 C
62 F62 5.5919 0.0022 -0.0001 F
63 F63 -5.5119 -0.0045 0.0016 F

Neutral SO_2 bridged $-OCH_3$ variant of Yamaguchi's hexacoordinated carbon compound (**S2**)

1 C1 0.0000 3.7678 -2.0129 C
2 C2 0.0000 2.5559 -1.3413 C
3 C3 0.0000 1.3248 -2.0298 C
4 C4 0.0000 1.4397 -3.4187 C
5 C5 0.0000 2.6425 -4.1077 C
6 C6 0.0000 3.8126 -3.3907 C
7 C7 0.0000 0.0000 -1.2936 C
8 C8 0.0000 -1.4397 -3.4187 C
9 C9 0.0000 -1.3248 -2.0298 C
10 C10 0.0000 -2.5559 -1.3413 C
11 C11 0.0000 -3.7678 -2.0129 C
12 C12 0.0000 -3.8126 -3.3907 C
13 C13 0.0000 -2.6425 -4.1077 C
14 H14 0.0000 4.6779 -1.4541 H
15 H15 0.0000 2.6431 -5.1782 H
16 H16 0.0000 4.7552 -3.8998 H
17 H17 0.0000 -4.6779 -1.4541 H
18 H18 0.0000 -4.7552 -3.8998 H
19 H19 0.0000 -2.6431 -5.1782 H
20 C20 0.0000 0.0000 1.2936 C
21 C21 -1.3248 0.0000 2.0298 C
22 C22 1.3248 -0.0000 2.0298 C
23 C23 -2.5559 0.0000 1.3413 C
24 C24 -1.4397 0.0000 3.4187 C
25 C25 1.4397 -0.0000 3.4187 C
26 C26 2.5559 -0.0000 1.3413 C
27 C27 -3.7678 0.0000 2.0129 C
28 C28 -2.6425 0.0000 4.1077 C
29 C29 2.6425 -0.0000 4.1077 C
30 C30 3.7678 -0.0000 2.0129 C
31 C31 -3.8126 0.0000 3.3907 C
32 H32 -4.6779 0.0000 1.4541 H
33 H33 -2.6431 0.0000 5.1782 H

34 C34	3.8126	-0.0000	3.3907	C
35 H35	2.6431	-0.0000	5.1782	H
36 H36	4.6779	-0.0000	1.4541	H
37 H37	-4.7552	0.0000	3.8998	H
38 H38	4.7552	-0.0000	3.8998	H
39 C39	0.0000	-0.0000	0.0000	C
40 O40	-2.5182	0.0000	-0.0127	O
41 O41	2.5182	0.0000	-0.0127	O
42 O42	0.0000	2.5182	0.0127	O
43 O43	-0.0000	-2.5182	0.0127	O
44 C44	0.0000	-3.6558	0.8630	C
45 H45	-0.8852	-4.2582	0.7036	H
46 H46	0.8852	-4.2582	0.7036	H
47 H47	0.0000	-3.2578	1.8645	H
48 C48	-3.6558	0.0000	-0.8630	C
49 H49	-4.2582	-0.8852	-0.7036	H
50 H50	-4.2582	0.8852	-0.7036	H
51 H51	-3.2578	0.0000	-1.8645	H
52 C52	-0.0000	3.6558	0.8630	C
53 H53	0.8852	4.2582	0.7036	H
54 H54	-0.8852	4.2582	0.7036	H
55 H55	-0.0000	3.2578	1.8645	H
56 C56	3.6558	-0.0000	-0.8630	C
57 H57	4.2582	0.8852	-0.7036	H
58 H58	4.2582	-0.8852	-0.7036	H
59 H59	3.2578	-0.0000	-1.8645	H
60 S60	0.0000	0.0000	-4.4295	S
61 S61	0.0000	0.0000	4.4295	S
62 O62	0.0000	1.3512	5.7136	O
63 O63	0.0000	-1.3512	5.7136	O
64 O64	-1.3512	-0.0000	-5.7136	O
65 O65	1.3512	-0.0000	-5.7136	O
